# Cathelicidin-HG Alleviates Sepsis-Induced Platelet Dysfunction by Inhibiting GPVI-Mediated Platelet Activation

**DOI:** 10.34133/research.0381

**Published:** 2024-06-05

**Authors:** Weichen Xiong, Jinwei Chai, Jiena Wu, Jiali Li, Wancheng Lu, Maolin Tian, Mohamed Amine Jmel, Johannes H. Ippel, Michail Kotsyfakis, Ingrid Dijkgraaf, Shuwen Liu, Xueqing Xu

**Affiliations:** ^1^ NMPA Key Laboratory for Research and Evaluation of Drug Metabolism, Guangdong Provincial Key Laboratory of New Drug Screening, School of Pharmaceutical Sciences, Southern Medical University, Guangzhou 510515, China.; ^2^ Institute of Parasitology, Biology Centre of the Czech Academy of Sciences, Branisovska 31, Budweis (Ceske Budejovice) 37005, Czech Republic.; ^3^ Department of Biochemistry, Cardiovascular Research Institute Maastricht (CARIM), Maastricht University, 6229 ER Maastricht, Netherlands.; ^4^ Institute of Molecular Biology and Biotechnology, Foundation for Research and Technology-Hellas, 70013 Heraklion, Crete, Greece.

## Abstract

Platelet activation contributes to sepsis development, leading to microthrombosis and increased inflammation, which results in disseminated intravascular coagulation and multiple organ dysfunction. Although Cathelicidin can alleviate sepsis, its role in sepsis regulation remains largely unexplored. In this study, we identified Cath-HG, a novel Cathelicidin from *Hylarana guentheri* skin, and analyzed its structure using nuclear magnetic resonance spectroscopy. The modulatory effect of Cath-HG on the symptoms of mice with sepsis induced by cecal ligation and puncture was evaluated in vivo, and the platelet count, degree of organ damage, and microthrombosis were measured. The antiplatelet aggregation activity of Cath-HG was studied in vitro, and its target was verified. Finally, we further investigated whether Cath-HG could regulate thrombosis in vivo in a FeCl_3_ injury-induced carotid artery model. The results showed that Cath-HG exhibited an α-helical structure in sodium dodecyl sulfate solution and effectively reduced organ inflammation and damage, improving survival in septic mice. It alleviated sepsis-induced thrombocytopenia and microthrombosis. In vitro, Cath-HG specifically inhibited collagen-induced platelet aggregation and modulated glycoprotein VI (GPVI) signaling pathways. Dot blotting, enzyme-linked immunosorbent assay, and pull-down experiments confirmed GPVI as the target of Cath-HG. Molecular docking and amino acid residue truncations/mutations identified crucial sites of Cath-HG. These findings suggest that GPVI represents a promising therapeutic target for sepsis, and Cath-HG may serve as a potential treatment for sepsis-related thrombocytopenia and thrombotic events. Additionally, identifying Cath-HG as a GPVI inhibitor provides insights for developing novel antithrombotic therapies targeting platelet activation mediated by GPVI.

## Introduction

Sepsis is a severe medical condition resulting from a dysregulated host response to infection that leads to organ dysfunction and poses a considerable threat to life [1]. It is currently recognized as a serious global health problem, being responsible for an average of about 20% of total deaths worldwide annually and posing a substantial burden to society [2]. Disseminated intravascular coagulation (DIC) is a fatal complication of sepsis [3]. DIC is a disorder of blood coagulation, characterized by the substantial aggregation of platelets and fibrin in the vasculature, ultimately leading to thrombosis, organ dysfunction, and death [4]. Thrombocytopenia is one of the common manifestations of DIC, which is usually caused by excessive platelet consumption, destruction, or reduced production [5]. Despite advances in clinical management, sepsis-related mortality remains high. Thus, it is crucial to develop novel therapeutic strategies for effectively preventing and treating sepsis-related to DIC and thrombocytopenia, which is decisive for improving patient survival and reducing the risk of multiple organ dysfunction syndrome.

Naturally occurring peptides have shown enormous potential as therapeutic agents [6–8]. Among them, the Cathelicidins, a class of natural antimicrobial peptides widely present in vertebrates, have been shown to possess multiple biological activities [9,10]. Amphibians are one of the important sources of Cathelicidins, and many Cathelicidins have been identified in this group [11–15]. Some studies have reported that Cathelicidins from amphibians exhibit antibacterial, anti-inflammatory, lipopolysaccharide (LPS) neutralizing, antioxidant, wound healing, and immunomodulatory properties [11,12,16,17]. These multifaceted activities are attributed to the remarkable structural diversity of the C-terminal antibacterial domain of Cathelicidins [18]. Therefore, the research and development of Cathelicidin family peptides will provide important insights and inspiration for discovering and developing novel natural medicines.

*Hylarana guentheri*, a sizeable species of Rana frog primarily distributed in southern China, has developed a defense mechanism that uses various biological molecules to combat several pathogens. Antimicrobial peptides derived from *H. guentheri* have been classified into the Esculentin-1, Temporin, and Brevinin-1 and -2 families [19–21]. However, no Cathelicidin peptide was identified in *H. guentheri*. Here, we report a Cathelicidin peptide, Cathelicidin-HG (Cath-HG), from the skin of *H. guentheri*. We first determined the mechanism by which Cath-HG affects platelet aggregation. Through a series of in vitro experiments, we found that Cath-HG specifically binds to the platelet glycoprotein VI (GPVI), thus inhibiting platelet activation and aggregation. In vivo experiments using a mouse model of sepsis showed that Cath-HG administration effectively prevented sepsis-related thrombosis and improved survival. In summary, our study demonstrated for the first time that Cath-HG, a natural peptide derived from *H. guentheri*, can effectively prevent sepsis-related thrombosis by targeting platelet GPVI. These findings highlight the potential of Cath-HG as a novel therapeutic agent for preventing and treating sepsis-related thrombosis.

## Results

### Structural characterization of Cath-HG

**Fig. 1.**
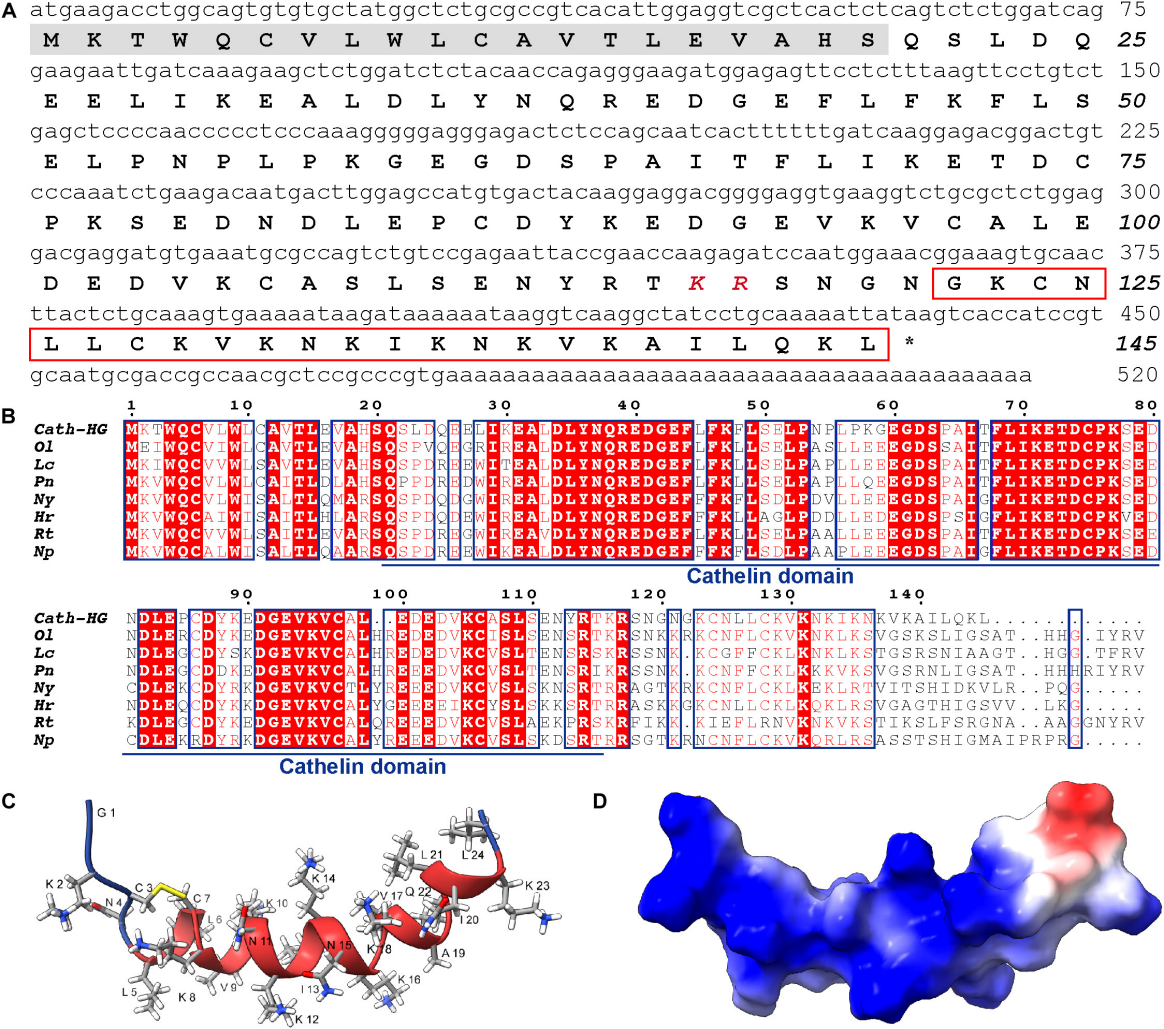
Identification and characterization of Cath-HG from *H. guentheri*. (A) The nucleotide sequence and predicted precursor sequence of Cath-HG. The presumed mature peptide is framed. The stop codon is represented by *. The signal peptide is gray shaded and is followed by a cathelin domain ending with KR residues (in red bold italics). The nucleotide and amino acid numbers are shown after the sequences. (B) Partial sequence similarity of Cath-HG precursor to homolog proteins from other amphibians (*Ol*, *O. livida*, AXR75914.1; *Lc*, *Lithobates catesbeianus*, QBC65431.1; *Pn*, *Pelophylax nigromaculatus*, QPL18198.1; *Ny*, *Nanorana yunnanensis*, AFX61592.1; *Hr*, *Hoplobatrachus rugulosus*, QQG31491.1; *Rt*, *Rana temporaria*, XP_040211152.1; *Np*, *Nanorana parkeri*, XP_018430778.1). Cathelin domain is indicated. The panel was generated with ESPript 3.0 (https://espript.ibcp.fr/ESPript/cgi-bin/ESPript.cgi). (C) Lowest–energy structure of Cath-HG with residue numbers and side chain. The secondary structure is represented in cartoon form, red indicates the α-helix, and yellow shows the Cys3–Cys7 disulfide bond that forms the N-terminal ring structure. The random coils are labeled with blue at the N- and the C-terminals. (D) Molecular surface of Cath-HG colored by electrostatic potential, with 2 colors describing the full range of positive (blue) and negative (red) charge across the surface.

A complete cDNA sequence encoding a Cathelicidin precursor was identified from a cDNA library of *H. guentheri* skin. As shown in Fig. 1A, the whole-length nucleotide sequence of the Cathelicidin is 520 bp. The deduced precursor contains 145 amino acids with a predicted signal peptide of 20 amino acids in the N-terminus. The National Center for Biotechnology Information CD-search showed that the secreted Cathelicidin had a typical cathelin domain, and a mature peptide (called Cath-HG) with 24 residues was predicted to be located at the C-terminus of the Cathelicidin according to the known frog Cathelicidins. The National Center for Biotechnology Information Basic Local Alignment Search Tool showed that the precursor of Cath-HG shares 81% identities (112/138) and 85% positives (118/138) with the precursor of OL-CATH2 from *Odorrana livida* [22]. Furthermore, the multiple sequence alignment showed that the Cath-HG precursor is highly similar to those from amphibians such as *O. livida*, *L. catesbeianus*, *P. nigromaculatus*, and *N. yunnanensis* (Fig. 1B). Although the C-terminal mature peptide of the Cath-HG precursor is partially structurally different from other amphibian-derived Cathelicidins, the mature Cath-HG shares a high sequence similarity in the N-terminal (2 to 15 residues), the disulfide-bonded pentapeptide ring, and the secondary structural features of frog Cathelicidin peptides previously studied (Fig. S1A and B). These results prompted us to further investigate the potential activity of Cath-HG. As shown in Fig. S2A, the purity and amino acid sequence of Cath-HG were identified by reversed-phase high-performance liquid chromatography (RP-HPLC) and matrix-assisted laser desorption ionization time-of-flight mass spectrometry (MALDI-TOF-MS).

Nuclear magnetic resonance (NMR) analysis of Cath-HG showed that Cath-HG dissolved in low-pH water adopts a random coiled structure, with the single disulfide bridge formed between Cys3 and Cys7 acting as the main structural constraint (data not shown). In sodium phosphate buffer at elevated alkaline pH, there was a slight preference for helix formation for several central residues after Cys7, an observation based on the detection of weak HN(i)-Ha(i-3) nuclear overhauser effects (NOEs) in this region typical of a classical α-helix fold. α-Helix formation was also evidenced by chemical shift index (CSI) values calculated from experimental Cath-HG chemical shifts of assigned Hα, Cα, and C_β_ amino acid resonances (Figs. S3 and S4A and C). However, the largest effect on peptide structure was after the addition of 55 mM deuterated sodium dodecyl sulfate-d^25^ (SDS-d^25^). The micellar environment created by SDS effectively stabilized the Cath-HG structure into a long α-helix capped by a protruding cyclic moiety at the N-terminal side (Fig. 1C and D). Delta CSI values determined for this SDS state of Cath-HG showed large increases indicating nearly pure α-helix formation between residues K8 and K23 (Fig. S4B and D). In addition, according to the assigned chemical shifts and NOE inter-residual peak patterns, the structure could be best described as an amphipathic helix, with most of the 8 lysine in the sequence grouped together. The helix segment most likely lies on top of the micelle sphere, leading to a slight bend in the helix backbone following more or less the curvature of the outer surface of the micelle particle. The peptide could also be depicted as having a ridge of hydrophobic side chains (L5, L6, V9, I13, V17, I20, L21, and L24) linearly aligned along the amphipathic peptide chain, which may well be used to bind extended thin aliphatic alkyl chains present in potential binding partners of Cath-HG, for instance in LPS. Comparing CSI values of free peptide at pH 7.0 with and without SDS demonstrated that ~20% of molecules in the free peptide relative to the SDS-stabilized form were able to adopt a central α-helix. The large structural change induced in SDS micelles was corroborated by nuclear overhauser effect spectroscopy (NOESY) of Cath-HG in SDS-d^25^, which shows many helical connectivity throughout the sequence (Fig. S5). The ^13^C-^1^H heteronuclear single-quantum coherence (HSQC) and natural-abundance ^15^N-^1^H HSQC spectra of Cath-HG (0.8 mM) displaying the Hα-Cα region are shown in Figs. S6 and S7. Unfortunately, the structural determination of the cyclic ring region by NMR was challenging. Relatively higher temperatures (minimum 37 °C) were required to narrow the line widths of proton resonances in the spectra, especially for residues C3 and L5 in the ring. These signals tended to broaden quickly as function of temperature, indicating a temperature-dependent dynamic process in the ring conformation.

### Cath-HG protects mice from sepsis-induced multiple organ dysfunction

**Fig. 2.**
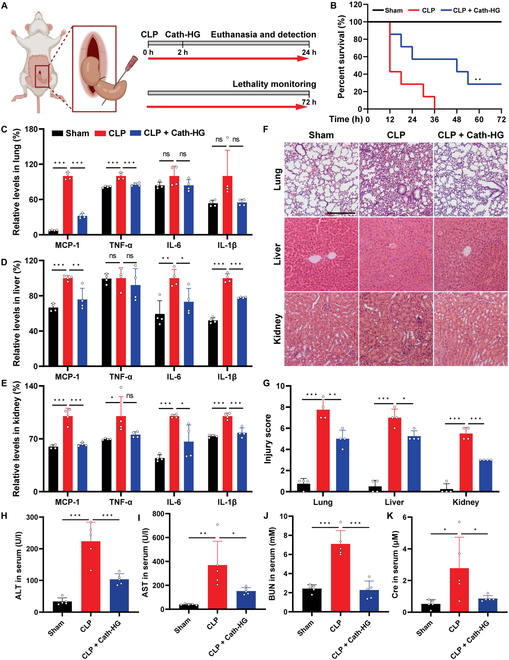
Cath-HG protects mice from sepsis-induced multiple organ dysfunction. (A) Experimental design involving Cath-HG treatment (5 mg/kg) to CLP mice. (B) Survival rate of CLP-induced septic mice after Cath-HG treatment (*n* = 7). (C to E) The concentrations of proinflammatory cytokines in lungs, liver, and kidneys measured by ELISA (*n* = 4). (F) Representative images of histopathological analysis of lungs, liver, and kidneys stained with H&E (scale bar = 200 μm). (G) Injury scores of lungs, liver, and kidneys in each group (*n* = 4). (H to K) The levels of alanine aminotransferase (ALT), aspartate aminotransferase (AST), blood urea nitrogen (BUN), and creatinine (Cre) in the serum (*n* = 5). Data are expressed as mean ± SD. *P < 0.05; **P < 0.01; ***P < 0.001; ns, not significant.

Cecal ligation and puncture (CLP) surgery causes polymicrobial infection in the peritoneal cavity of mice, leading to the generation of sepsis [23]. The protective effect of Cath-HG on CLP-induced sepsis was investigated in mice based on the structure similarity of Cath-HG with Cath-MH, an antiseptic Cathelicidin with multiple mechanisms of action (Fig. 2A and Fig. S1B) [12,24]. As shown in Fig. 2B, Cath-HG markedly attenuated septic symptoms and increased the survival of CLP-treated mice. Specifically, the survival rates of sham, CLP, and Cath-HG-treated mice were 100%, 0%, and 29% 72 h after operation, respectively (Fig. 2B). Furthermore, CLP significantly increased monocyte chemoattractant protein-1 (MCP-1), tumor necrosis factor-α (TNF-α), interleukin-6 (IL-6), and IL-1β protein levels in the lungs, liver, kidneys, and serum compared with the sham group while Cath-HG significantly reversed the increase in these proinflammatory cytokines in CLP septic mice (Fig. 2C to E and Fig. S8). In addition, hematoxylin and eosin (H&E) staining analysis of the organs showed a protective effect for Cath-HG in CLP-induced sepsis. Compared with sham group, CLP mice displayed severe pathological damage in their lungs, liver, and kidneys, such as thicker alveolar walls, more intense inflammatory infiltrates, alveolar destruction, and alveolar space obliteration in the lungs. Again, Cath-HG ameliorated this pathological damage (Fig. 2F and G). Notably, CLP surgery also significantly increased serum levels of liver and kidney function markers, including alanine aminotransferase, aspartate aminotransferase, blood urea nitrogen, and creatinine, indicating organ damage. The administration of Cath-HG effectively reduced these elevated markers (Fig. 2H to K). Collectively, these results indicated that Cath-HG improved multiple organ dysfunction in CLP-treated mice.

### Cath-HG attenuates excessive platelet activation in septic mice

**Fig. 3.**
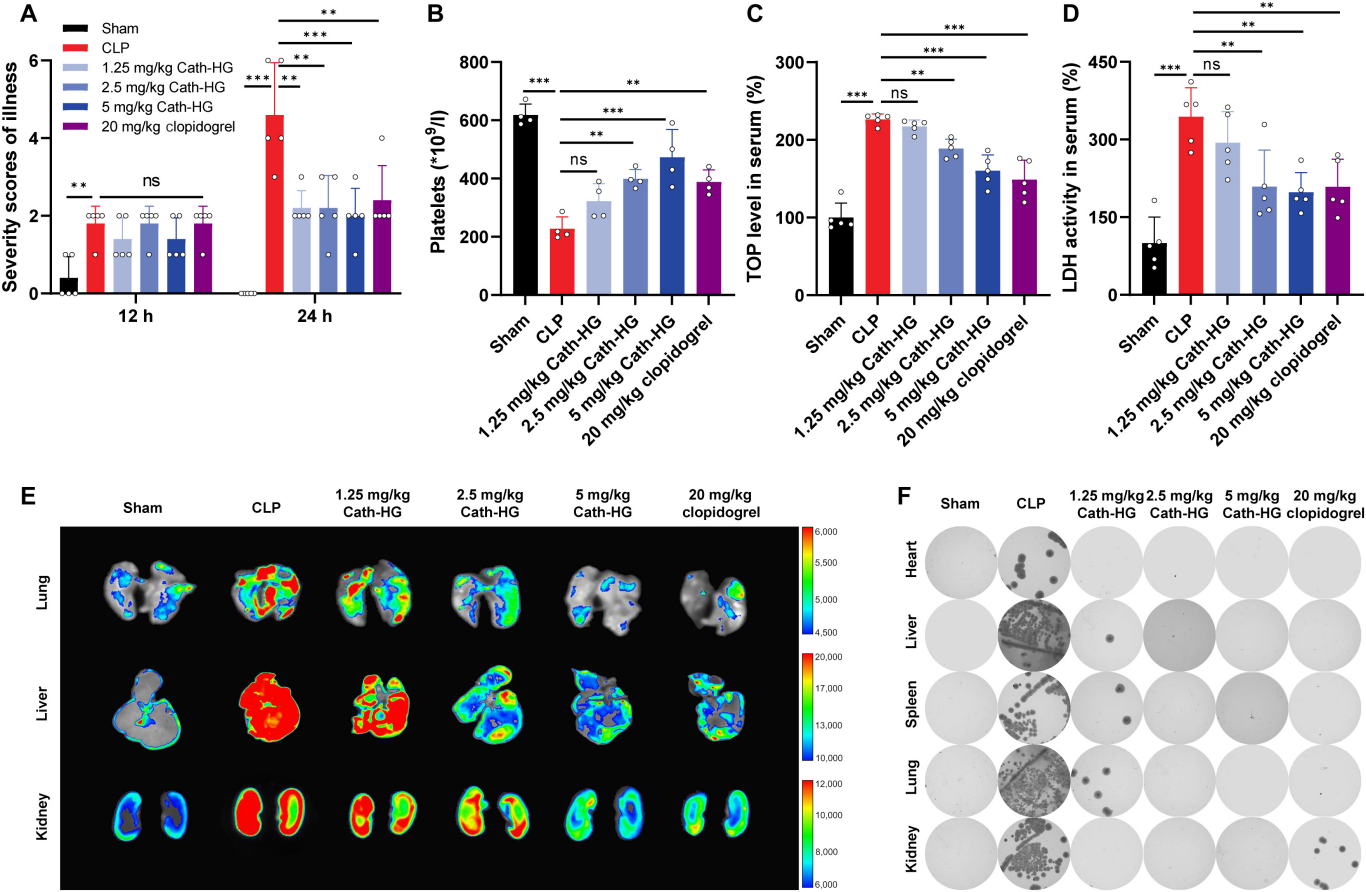
Cath-HG attenuates excessive platelet aggregation in septic mice. (A) Severity scores of illness for each group at 12 and 24 h after CLP. Mice were intraperitoneally injected with Cath-HG (1.25, 2.5, and 5 mg/kg) or clopidogrel (20 mg/kg) 2 h after CLP (*n* = 4). (B to D) Platelet counts, TOP levels, and LDH activity in serum of mice in each group (*n* = 4). (E) Microvascular thrombosis in the lungs, liver, and kidneys detected by fluorescent microbeads through imaging. (F) Bacterial loads in the heart, liver, spleen, lung, and kidney. Data are expressed as mean ± SD. **P < 0.01; ***P < 0.001; ns, not significant.

Excessive aggregation of platelets induced by sepsis can lead to thrombocytopenia and DIC [5]. Therefore, after confirming the therapeutic effect of Cath-HG on sepsis, we further tested its effects on platelets in septic mice at different concentrations. First, we assessed the severity of illness in the sham and model groups after CLP. The sham group had a few ruffled furs at 12 h, and the score decreased to 0 at 24 h. The severity of illness was similar among groups 12 h after CLP. However, mice receiving only CLP had higher severity scores after 24 h compared with groups treated with different concentrations of Cath-HG (Fig. 3A). The platelet count assay showed that CLP-induced sepsis in mice resulted in a severe fall in platelet count at 24 h (Fig. 3B). Cath-HG treatment significantly attenuated the CLP-induced decrease in platelet count. Similar effects have been observed with the antiplatelet aggregation drug clopidogrel. Furthermore, the levels of thrombopoietin (TPO) in serum were markedly elevated in CLP mice, suggesting that the reduction in platelet counts initiates a compensatory feedback mechanism aimed at stimulating platelet production (Fig. 3C) [25,26]. Likewise, the levels of TPO in serum were significantly decreased in the Cath-HG treatment group in a concentration-dependent manner.

The activity of lactate dehydrogenase (LDH), which is an important index reflecting the degree of end-organ damage caused by microvascular thrombosis and tissue hypoxia, was significantly increased in CLP mice but significantly decreased after Cath-HG or clopidogrel treatment (Fig. 3D) [27]. Microvascular thrombosis in lungs, liver, and kidneys was further detected by fluorescence imaging. Fluorescent beads can accumulate in embolized vessels, resulting in increased fluorescent signal. The lungs, liver, and kidneys of sham group showed only faint fluorescence, while strong fluorescent signals were observed in CLP mice, indicating that sepsis caused the formation of thrombus in the organs (Fig. 3E). Cath-HG and clopidogrel treatment reduced the fluorescence levels of lungs, liver, and kidneys. Thrombocytopenic septic mice also exhibit organs bacterial growth [28]. Therefore, the bacterial load of viscera was examined. At 24 h after CLP, mice showed substantial bacterial load in heart, liver, spleen, lung, and kidney, which was reduced by Cath-HG treatment. At the Cath-HG concentrations of 2.5 and 5 mg/kg, no bacteria have been observed (Fig. 3F). Notably, the antiplatelet drug clopidogrel also reduced the bacterial burden in the organs of CLP mice.

Furthermore, platelets have been demonstrated to stimulate neutrophils to release neutrophil extracellular traps (NETs) during sepsis [29]. The examination of NETs components, including myeloperoxidase-DNA and neutrophil elastase, revealed a significant increase in neutrophil elastase levels in the lungs and elevated myeloperoxidase-DNA levels in the serum of septic mice (Fig. S9). Following treatment with Cath-HG, the levels of NETs were markedly reduced. Therefore, these observations indicate an obvious protective effect of Cath-HG on excessive platelet activation in septic mice.

### Cath-HG inhibits collagen-induced platelet aggregation

**Fig. 4.**
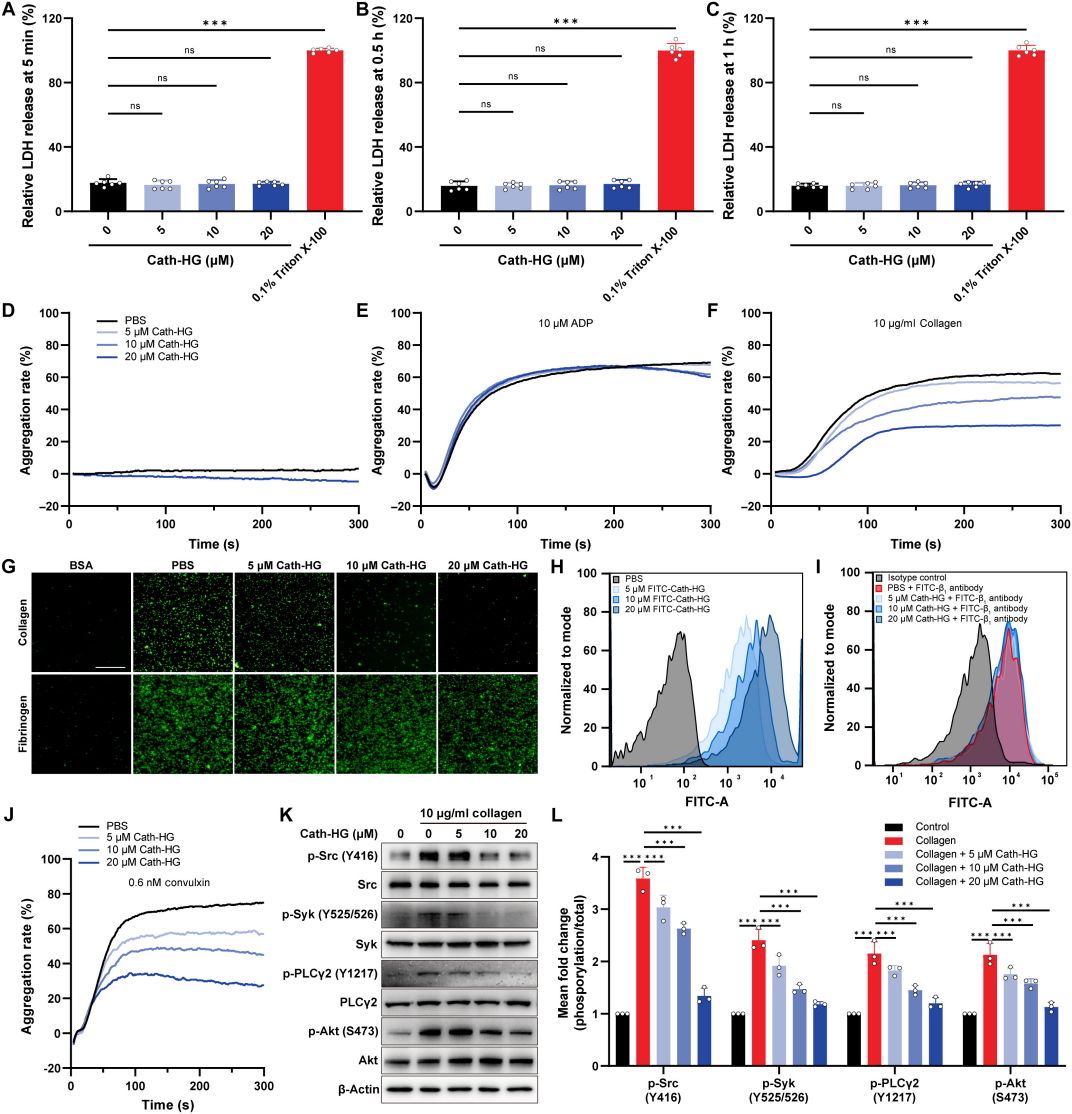
Cath-HG inhibits collagen-induced platelet aggregation. (A to C) Release of LDH from washed platelets after Cath-HG (5, 10, and 20 μM) treatment for 5 min, 0.5 h, and 1 h (*n* = 5). (D) Platelet aggregation of PRP after Cath-HG (20 μM) treatment for 5 min. (E and F) Platelet aggregation of PRP induced by ADP (10 μM) and collagen (10 μg/ml) after Cath-HG (5, 10, and 20 μM) treatment for 5 min. (G) Spreading of platelets (green fluorescence) on collagen and fibrinogen (scale bar = 80 μm). (H) Flow cytometry analysis of Cath-HG (5, 10, and 20 μM) binding to platelets. (I) Flow cytometry analysis of the effect of Cath-HG (5, 10, and 20 μM) on the binding of platelet and β_1_ antibody. (J) Platelet aggregation of PRP induced by convulxin (0.6 nM) after Cath-HG (5, 10, and 20 μM) treatment for 5 min. (K) Immunoblot and (L) corresponding quantification analysis of phosphorylation of Src, Syk, PLCγ2, and Akt in washed platelets stimulated with collagen after 5-min treatment with or without Cath-HG (5, 10, and 20 μM). β-Actin was used as a loading control (*n* = 3). Data are expressed as mean ± SD. ***P < 0.001; ns, not significant.

Considering the beneficial effect of Cath-HG on platelets in sepsis, we further investigated the effect of Cath-HG on platelets from normal mice in vitro. We first evaluated the cytotoxicity of Cath-HG, and the results showed that Cath-HG did not cause extra LDH release from platelets and platelet aggregation in platelet-rich plasma (PRP) (Fig. 4A to D). In platelet aggregation inhibition assays, Cath-HG inhibited collagen rather than adenosine diphosphate (ADP)-induced platelet aggregation (Fig. 4E and F). No inhibitory effect on platelet aggregation was observed with other tested Cathelicidins derived from amphibians (Fig. S10). In line with platelet aggregation inhibition assays, Cath-HG did not affect platelet adhesion and spreading on fibrinogen but inhibited the adhesion between platelets and collagen in a concentration-dependent manner (Fig. 4G). This led us to speculate that Cath-HG specifically inhibits the binding between collagen and its receptor on platelets.

Pursuing this, fluorescein isothiocyanate (FITC)-labeled Cath-HG was incubated with washed platelets to define its target. As shown in Fig. 4H, the fluorescence intensity of platelets was increased with the increasing concentrations of Cath-HG, indicating that Cath-HG targets platelets instead of collagen. Furthermore, at various concentrations, Cath-HG did not affect the binding of platelets to β_1_ antibody, indicating that the target of Cath-HG is not α_2_β_1_ (Fig. 4I). However, Cath-HG concentration-dependently inhibited platelet aggregation induced by the GPVI-specific agonist convulxin, and it inhibited 63.2% of platelet aggregation at a concentration of 20 μM, implying that Cath-HG inhibits GPVI activity (Fig. 4J).

Western blot was used to detect GPVI downstream pathway proteins in collagen-activated platelets. The results showed that Cath-HG did not lead to the activation of the GPVI downstream signaling pathway (Fig. S11). After using the Src inhibitor dasatinib, the phosphorylation level of collagen-activated GPVI downstream pathway proteins was completely inhibited. Similarly, Cath-HG also significantly inhibited collagen-induced phosphorylation levels of Src, Syk, PLCγ2, and Akt (Fig. 4K and L). These findings further suggest that Cath-HG may target GPVI on platelets to inhibit collagen-induced platelet aggregation.

### Cath-HG directly targets to collagen receptor GPVI

**Fig. 5.**
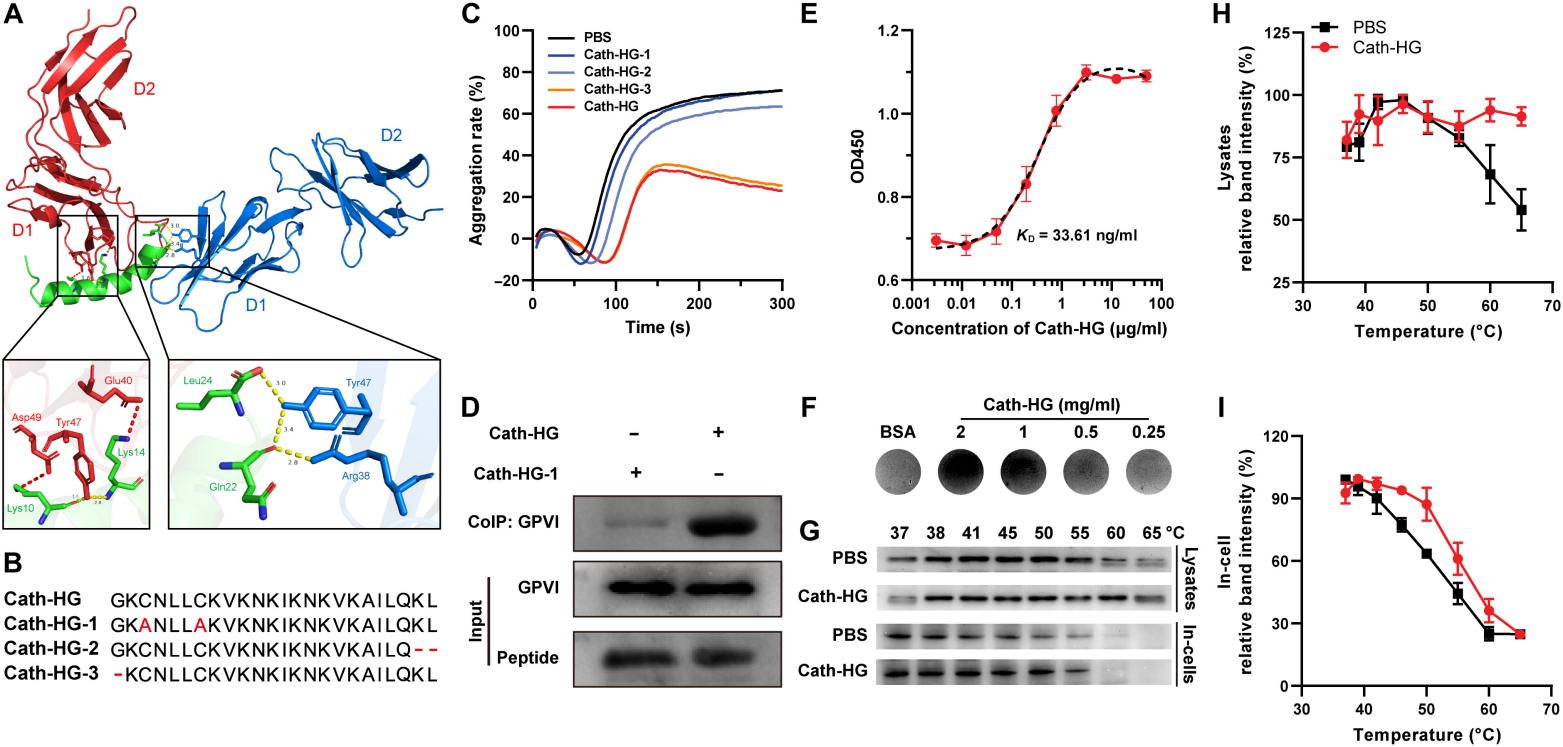
Cath-HG directly targets to collagen receptor GPVI. (A) The 3D image of molecular docking showing Cath-HG (green) binding to the D1 domain of GPVI (red and blue). Lys10, Lys14, Leu24, and Gln22 of Cath-HG are predicted to interact with Arg38, Glu40, Try47, and Asp49 of GPVI. Red dotted lines indicate hydrophobic interactions, and yellow dotted lines indicate hydrogen bonds. (B) Sequence alignment of Cath-HG and its modified peptides. (C) Platelet aggregation of PRP induced by collagen (10 μg/ml) after Cath-HG or its modified peptides (20 μM) treatment for 5 min. (D) Pull-down analysis of Cath-HG selectively binding to GPVI. (E and F) Binding reaction between Cath-HG and GPVI. The platelet lysate was incubated with the indicated concentrations of Cath-HG immobilized in a 96-well plate or PVDF membranes before the contents of GPVI was detected by anti-GPVI primary antibody with corresponding ELISA (E) and dot blot (F) method, respectively (*n* = 3). (G to I) CETSA. The platelet or platelet lysate were incubated with Cath-HG (50 μM) at room temperature for 30 min and then the mixtures were heated for 3 min at indicated temperatures (37 to 65 °C) before the contents of GPVI were detected by Western blot (*n* = 3). Data are mean ± SD.

To offer additional direct positive evidence for the binding of Cath-HG and GPVI, we performed target validation in different experiments. First, the binding site of Cath-HG on GPVI (PDB ID: 5OU8) was investigated using molecular docking. Docking simulations revealed that Cath-HG bound to the binding pocket of the D1 domain, which is the binding site for collagen on GPVI (Fig. 5A). Asp49 and Glu40 of the GPVI A chain (red) formed hydrophobic interactions with Lys10 and Lys14 of Cath-HG (green), respectively. Tyr47 of the GPVI A chain formed 2 hydrogen bonds with Lys10 and Lys14 of Cath-HG. Try47 and Arg38 of GPVI B chain (blue) formed 3 hydrogen bonds with Leu24 and Gln22 of Cath-HG. These docking results prompted us to truncate or mutate Cath-HG to verify or explore the key binding sites and finally synthesized the modified peptides Cath-HG-1, -2 and -3 (Fig. 5B and Fig. S2B to D). Notably, Cath-HG-3 has similar inhibitory activity on collagen-induced platelet aggregation with Cath-HG, while Cath-HG-1 and -2 lost their activity, implying that the disulfide bond and N-terminal residues play an important role in maintaining the activity of Cath-HG (Fig. 5C).

Next, a pull-down assay was performed using biotinylated Cath-HG with platelet lysates. As expected, after harsh washing conditions, the eluted parts of the Cath-HG group showed a strong GPVI signal, whereas Cath-HG-1 did not (Fig. 5D). Consistent with these results, both enzyme-linked immunosorbent assay (ELISA)-based binding assay and dot blotting analysis showed a tight and specific interaction between Cath-HG and GPVI. The ELISA experiments also revealed that the potent *K*_D_ of Cath-HG to GPVI was 33.61 ng/ml (Fig. 5E and F). Moreover, Cellular thermal shift assay (CETSA) experiments were performed to additionally support the direct interaction of Cath-HG with GPVI. The heat-pulse results showed that, as the temperature increased, GPVI in platelets or lysates gradually denatured, exhibiting a decrease in content. Notably, Cath-HG protected GPVI in both cells and lysates from temperature-dependent denaturation (Fig. 5G to I). In combination, the findings suggest that Cath-HG can directly bind to GPVI, and disulfide bonds and N-terminal residues are important for the binding of Cath-HG to GPVI.

### Cath-HG inhibits thrombus formation in vivo

**Fig. 6.**
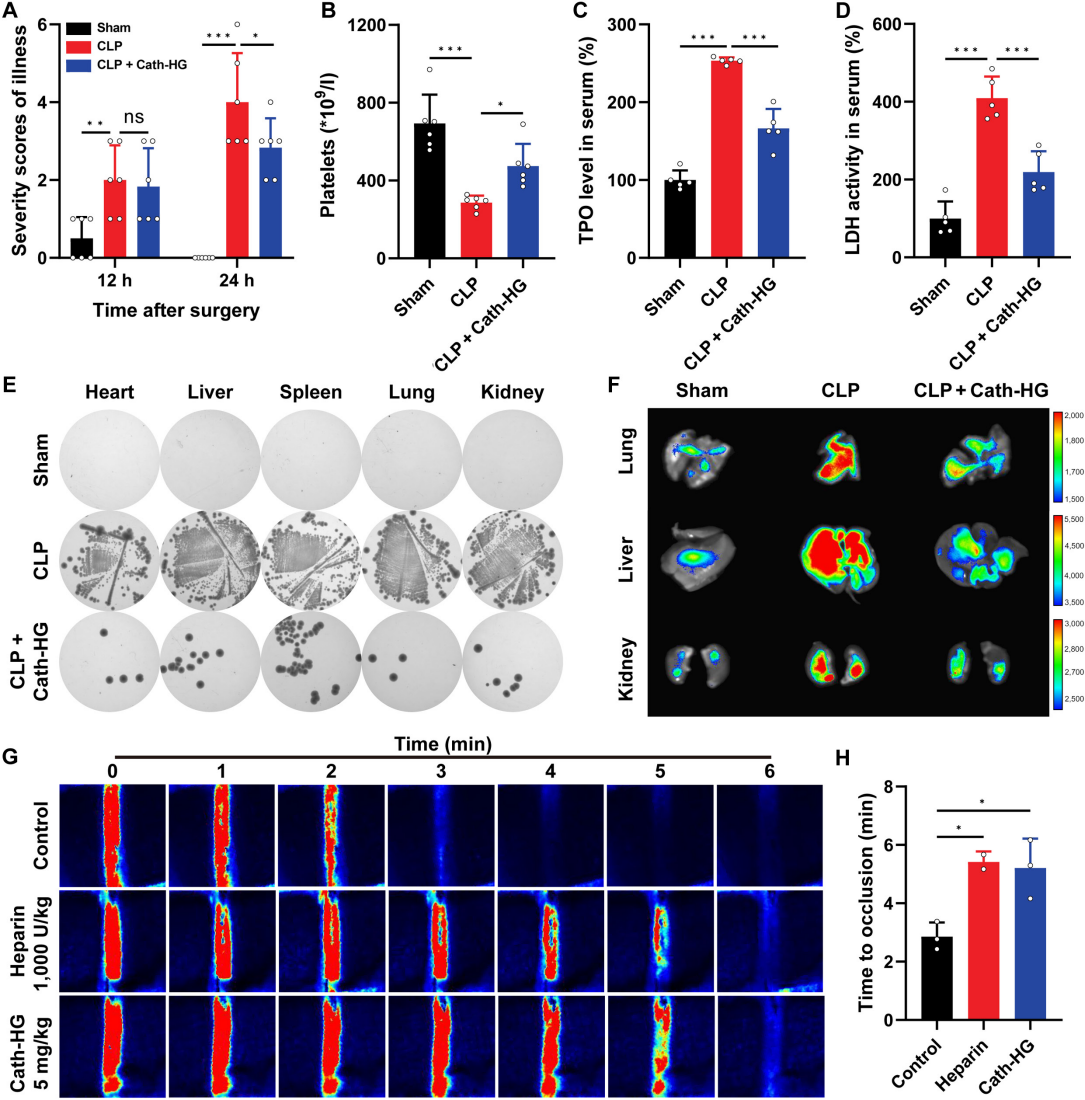
Cath-HG inhibits thrombus formation in vivo. Cath-HG (5 mg/kg) was intravenously injected into mice after 2 h of CLP, and the severity scores of illness (A), platelet count (B), TPO level (C), and LDH level (D) in the serum were determined (*n* = 5 to 6). (E) Lysogeny broth agar plate culture detecting bacterial load in the heart, liver, spleen, lungs, and kidneys. (F) Fluorescence imaging detecting microvascular thrombosis in the lungs, liver, and kidneys of CLP mice after intravenous injection of Cath-HG (5 mg/kg). (G) Representative images of carotid artery blood flow in FeCl_3_-treated mice. The mice received intravenous injections of 0.9% saline (*w/v*), heparin (1,000 U/kg), or Cath-HG (5 mg/kg) 30 min prior to injury and were subsequently monitored using a laser speckle flow imaging system. Red indicates blood flow, while blue-black areas indicate background. (H) Complete occlusion time of carotid arteries in FeCl_3_-injured mice after intravenous injection of samples (*n* = 3). Data are expressed as mean ± SD. *P < 0.05; **P < 0.01; ***P < 0.001; ns, not significant.

Finally, intravenous injection of Cath-HG was used to further validate its inhibitory effect on thrombus formation in vivo. Similar to the results of intraperitoneal injection, intravenous injection of Cath-HG also alleviated the symptoms of septic mice, increased the platelet count, and reduced the TPO level, which indicated that Cath-HG ameliorated thrombocytopenia in septic mice (Fig. 6A to C). At the same time, the decrease in LDH level, the reduction of organ bacterial load, and the decrease in thrombus formation indicated that Cath-HG reduced the damage of thrombus formation to organs (Fig. 6D to F and Fig. S9). In order to provide a more comprehensive characterization of the inhibitory effect of Cath-HG on in vivo thrombus formation, a FeCl_3_-induced arterial thrombosis model was also applied. As shown in Fig. 6G and H, carotid blood flow could not be detected in control mice within 3 min after injury (mean occlusion time 2.9 ± 0.3 min), while intravenous injection of heparin or Cath-HG significantly increased blood flow and prolonged the mean occlusion time to 5.4 ± 0.3 min and 5.2 ± 0.6 min, respectively. These results once again strongly indicate that Cath-HG effectively inhibits thrombus formation in vivo.

## Discussion

Sepsis is a critical condition characterized by systemic inflammatory response and organ dysfunction [30]. Despite advances in antimicrobial therapies, it remains a major global health burden [31]. In response to diverse survival challenges, amphibian skin synthesizes a highly diverse range of defensive molecules, comprising peptides that exhibit numerous pharmacological activities, including but not limited to antimicrobial, antiviral, antioxidant, and anti-inflammatory effects [32–35]. Our study focused on Cath-HG, a novel Cathelicidin from *H. guentheri*, highlighting its role in sepsis by targeting the platelet collagen receptor GPVI. This action inhibits platelet overactivation, reducing organ damage and improving survival in septic mice. This suggests that inhibiting platelet activation could be a novel therapeutic strategy for sepsis, with Cath-HG being a promising candidate.

Platelets are key in innate immunity, and their overactivation in sepsis can promote microthrombosis and exacerbate inflammatory response, resulting in thrombocytopenia, DIC, and multiple organ dysfunction, ultimately leading to death [3,36–38]. Evidence shows that antiplatelet drugs like clopidogrel can mitigate sepsis-induced inflammation and organ dysfunction [39–41]. Cath-HG was found to restore platelet count and reduce organ damage by reducing microthrombosis in septic mice. Thrombocytopenia has also been reported to impair host defenses leading to increased bacterial load in organs [28]. Consistent with this, Cath-HG treatment substantially reduces bacterial load in organs. Encouragingly, administration of the antiplatelet aggregation drug clopidogrel also reduced bacterial load in organs, which strongly suggests that the reduction in bacterial load is not solely due to the potential antibacterial effect of Cath-HG as a member of the Cathelicidin family antimicrobial peptides. It is worth noting that treatments with antibiotics, despite their stronger antimicrobial effects, have been reported to have limited therapeutic efficacy in sepsis, or in some cases, can even worsen organ damage [42–44]. Therefore, the in vivo study results of Cath-HG have uncovered its substantial potential in treating sepsis, surpassing mere antimicrobial action, which has stimulated our enthusiasm to further investigate its mechanisms of action.

GPVI, an immunoglobulin superfamily member, is a type I transmembrane glycoprotein that covalently binds to the Fc receptor γ chain to regulate signal responses by facilitating signal transduction [45]. The crosslinking of GPVI induces phosphorylation of the 2 cytoplasmic immunoreceptor tyrosine-based activation motifs in the Fc receptor γ chain by the Src family tyrosine kinases [46]. This event subsequently triggers the recruitment and phosphorylation of Syk, leading to the activation of linker for activation of T cells. Subsequently, linker for activation of T cells serves as a platform for downstream phosphorylation of PLCγ2 and activation of the classical phosphoinositide 3-kinase-Akt signaling pathway [46]. Cath-HG effectively inhibits platelet aggregation and reduces phosphorylation of signaling proteins downstream of GPVI. Molecular docking and subsequent experiments confirmed the binding of Cath-HG to GPVI. Most notably, FeCl_3_ diffuses through the vascular wall, causing endothelial denudation and exposing the subendothelial matrix, ultimately leading to thrombus formation, in which GPVI plays a critical role [47]. Cath-HG significantly prolongs the time of FeCl_3_-induced carotid artery thrombus formation in mice. These results support the role of Cath-HG in regulating platelet activation through GPVI.

The complex role of GPVI in sepsis has been explored in several studies, providing preliminary insights into its function. Claushuis and colleagues [48] reported that both GPVI-depleted and GPVI^-/-^ mice showed increased bacterial growth in the lungs in a *Klebsiella pneumoniae*-derived sepsis model. In contrast, Burkard et al. [49] showed that GPVI-deficient mice as well as anti-GPVI-treated WT mice were markedly protected in the LPS-induced acute lung injury model. These findings highlight the diverse roles of GPVI in sepsis, suggesting that GPVI acts as a double-edged sword: on one hand, facilitating bacterial clearance, and on the other, contributing to severe thrombosis and inflammation. Based on our study, the inhibition of GPVI by Cath-HG avoids organ damage caused by thrombosis and the inflammatory response caused by neutrophil activation in sepsis. Furthermore, the additional antibacterial activity of Cath-HG offset the reduced bacterial clearance capability caused by GPVI inhibition. These synergistic effects give Cath-HG considerable therapeutic potency in the treatment of sepsis. Since human sepsis is usually triggered by bacterial infection, the therapeutic achievements of Cath-HG in sepsis imply that the combined strategy of anti-GPVI therapy and antibacterial agents provides a novel reference for the treatment of human sepsis.

The safety assessment indicates that Cath-HG is nontoxic, and moreover, it contains only 24 amino acids, which is far less than other peptides in the same family. This highlights its advantages of safety and low-cost synthesis, paving the way for its clinical development. However, our research has several limitations that need to be addressed in future studies. Firstly, we did not investigate the therapeutic effects of Cath-HG treatment at longer time points after CLP surgery, which could provide valuable insights into its efficacy in more complex and clinically relevant settings. Secondly, considering that Cath-HG belongs to the Cathelicidin family, it may have additional antibacterial, anti-inflammatory, and other bioactive properties. This multifunctional nature suggests that Cath-HG might serve as a versatile therapeutic agent for sepsis, targeting multiple aspects of the complex pathophysiology of the condition. It is important to emphasize that these limitations do not diminish the strength of conclusions drawn from our study, as the findings clearly demonstrate the inhibitory effect of Cath-HG on platelet activation and its potential as a promising strategy for sepsis treatment.

In summary, our study identifies and characterizes a novel Cathelicidin, Cath-HG, from the skin of *H. guentheri*, which effectively alleviates sepsis-induced platelet dysfunction and organ damage by specifically inhibiting GPVI-mediated platelet activation. Our findings, for the first time, establish GPVI as a promising therapeutic target for sepsis and Cath-HG as a potential treatment for sepsis-related thrombocytopenia and thrombotic events. The identification of Cath-HG as a GPVI inhibitor also provides insights for the development of novel antithrombotic therapies targeting GPVI-mediated platelet activation.

## Materials and Methods

### Reagents

MCP-1, TNF-α, IL-6, and IL-1β ELISA assay kits, EZ-Link Desthiobiotinylation and Pull-Down Kit, as well as fluorescent beads, were obtained from Invitrogen (CA, USA). TPO ELISA assay kit was purchased from Elabscience (Wuhan, China). The LDH Cytotoxicity Assay Kit was obtained from Beyotime (Shanghai, China). Collagen and ADP were purchased from Hyphen-BioMed (Neuville-sur-Oise, France). The radioimmunoprecipitation assay cell lysis buffer was obtained from KeyGEN (Jiangsu, China). The primary antibodies against p-Src (Y416), Src, p-Syk (Y525/526), Syk, p-PLCγ2 (Y1217), PLCγ2, p-Akt (S473), Akt, and β-Actin, as well as the secondary rabbit antibodies, were purchased from Cell Signaling Technology (MA, USA). Anti-GPVI antibody was purchased from HUABIO (Hangzhou, China).

### Cloning and characterization of Cath-HG cDNA

The *H. guentheri* skin transcriptome was constructed following our previous study, and the cDNA encoding Cath-HG was obtained from it [34]. The signal peptide was analyzed using the SignalP 6.0 (https://services.healthtech.dtu.dk/service.php?SignalP) [50]. The physicochemical properties of Cath-HG were forecasted and analyzed through the application of the ExPASy Bioinformatics Resource Portal (http://www.expasy.org/tools/) and the blastp suite (https://blast.ncbi.nlm.nih.gov/blast.cgi).

### Peptide synthesis

The mature peptide Cath-HG, FITC-labeled Cath-HG, and its analogs were commercially prepared by SciLight (Beijing, China). Following synthesis, the crude peptide underwent purification on an Inertsil ODS-SP (C-18) RP-HPLC column. The resulting peptide, boasting a purity of 98.4%, was then combined, lyophilized, and subjected to further analysis via MALDI-TOF-MS before storage at −20 °C until use.

### NMR spectroscopy

Cath-HG was investigated by NMR spectroscopy using various sample preparations: 0.68 mM Cath-HG in deionized water (pH 3.5); 0.8 mM Cath-HG in 50 mM phosphate-buffered saline (PBS) (pH 5.8 and 7.0), and 0.8 mM Cath-HG in SDS micelles in the presence of 50 mM NaPi buffer (pH 7.0) with or without a final concentration of 55 mM fully deuterated SDS-d^25^ (Merck, Darmstadt, Germany) detergent at different temperatures. All NMR spectra were recorded on a Bruker Avance III HD 700-MHz spectrometer (Rheinstetten, Germany) operating at a frequency of ^1^H 700 MHz and equipped with a TCI CryoProbe. Real sample temperatures inside the NMR probe were calibrated by an external thermocouple inserted into a water-filled dummy NMR tube. NMR spectra were processed using Topspin3.2 (Bruker, Rheinstetten, Germany), and Sparky 3.114 (https://www.cgl.ucsf.edu/home/sparky/) was used for manual assignment and NOE analysis. Various standard 1-dimensional and gradient-enhanced 2-dimensional (2D) experiments from the Bruker pulse library were applied to create sequential assignments of the peptide: composite-pulse decoupling in the presence of scalar interactions (DIPSI, 70-ms mixing time), NOESY (50 to 350-ms mixing time), rotating frame overhauser effect spectroscopy (ROESY, 150-ms mixing time), and natural HSQC abundance ^13^C-^1^H, HSQC-DIPSI 70 ms, HSQC ^15^N-^1^H heteronuclear spectra. Assigned chemical shifts were collected for all 3 different Cath-HG solutions. 2D spectra of Cath-HG in 55 mM SDS-d^25^ were analyzed in detail at 37 °C to solve the solution structure of the peptide. Complete information about structure determination and analysis of Cath-HG is provided in the Supplementary Materials.

### Animals

Male BALB/c mice weighing between 18 and 25 g and aged 6 to 8 weeks were procured from the Laboratory Animal Center of Southern Medical University (Guangzhou, China). The mice were housed in a specific pathogen-free environment and subjected to standard laboratory conditions, including a 12-h light/dark cycle, 55% to 65% relative humidity at 22 °C. The mice were acclimatized for a minimum of 1 week and were given free access to food and water before the formal experiment. The animal care procedures and experimental manual were implemented in accordance with relevant guidelines and were authorized by the Ethics Committee for Experimental Animals at Southern Medical University (No. L2018274).

### CLP model

CLP-induced sepsis was performed based on our previous method with minor modifications [33]. Briefly, under sterile conditions, the cecum of anesthetized mice was exposed and tightly ligated approximately 1 cm from the end. Then, the cecum was punctured twice at different mid-points with a 22-gauge needle, gently squeezed to extrude a small amount of fecal matter, returned into the peritoneal cavity which was sutured. Finally, the mice underwent resuscitation through the administration of 1 ml of preheated physiological saline via subcutaneous injection. At 2 h postsurgery, the mice were randomly administered different concentrations of Cath-HG or an equal volume of physiological saline via intraperitoneal or tail vein injection. Clopidogrel (20 mg/kg) was administered as a positive control, and blank control mice underwent sham surgery and received saline. All mice were monitored for 72 h after the induction of CLP. Another group of mice underwent the same procedure described above. Twenty-four hours after the surgery, the mice were euthanized, and blood, heart, liver, spleen, lung, and kidney samples were collected for subsequent analysis.

### Severity score of illness

To assess the severity of sepsis, the state of the mice was evaluated using the scoring system described by Gonçalves et al. [51] as follows: appearance (0 = smooth fur, 1 = ruffled fur), eye aspect (0 = normal, 1 = secretions), activity (0 = active, 1 = lethargic, 2 = moribund), consciousness (0 = alert, 1 = sleepy), and respiration (0 = normal rapid breaths, 1 = slow-labored breaths). Higher scores reflect increased illness severity. Evaluations were performed at 12 and 24 h after surgery.

### Detection of inflammatory factors

In order to measure the levels of inflammatory cytokines, ELISA was applied to detect the concentration of MCP-1, TNF-α, IL-6, and IL-1β in the tissues. Briefly, the lungs, liver, and kidneys of the mice were homogenized in ice-cold PBS and then centrifuged at 10,000× *g* for 15 min at 4 °C. The resulting supernatants were collected and analyzed using commercial ELISA kits according to the manufacturer’s instructions.

### Histology

Sections (4 μm) of the lung, liver, and kidney were stained with H&E and scored for histopathological injuries as previously described [12]. The assessment criteria encompassed vascular congestion, edema, and leukocyte infiltration in the lung; periportal inflammation, hepatocyte necrosis, and immune cell infiltration in the liver; as well as vacuolization and cast formation in the kidney. Each criterion was assigned a score from 0 to 3, representing “none”, “mild”, “moderate”, and “severe”, respectively. The aggregate injury score was calculated as the sum of all factors, with a maximum value of 9.

### Fluorescence imaging of microvascular thrombosis

Fluorescence imaging of microvascular thrombosis was performed using 2.5-μm fluorescent beads to label the clots in the organ microvasculature [52,53]. Briefly, the fluorescent beads were diluted to 1.2 × 10^6^ beads/ml in sterile physiological saline and injected into the tail vein of the mice at a volume of 100 μl 3 h after surgery. The collected tissues were fixed with 4% paraformaldehyde and imaged using an In-Vivo Imaging System Fx Pro (Bruker, MA, USA). As the fluorescent beads were trapped in the thrombotic vessels, the fluorescence intensity reflected the severity of the thrombosis.

### Bacterial loads

To determine bacterial loads in each organ, the heart, liver, spleen, lung, and kidney were homogenized in 10-fold sterile physiological saline and then further diluted 10-fold. The diluted samples were then plated onto lysogeny broth agar and incubated at 37 °C for 16 h to determine the number of colony-forming units [54].

### Platelet count and serum cytokines

For platelet count determination, blood was collected in EDTA and analyzed using a PE-6800 Fully Auto Hematology Analyzer (Prokan, Shenzhen, China). For the measurement of serum cytokines, whole blood was collected without anticoagulant and left at room temperature for 1 h before centrifugation at 2,000× *g* for 30 min to separate the serum. TPO levels were determined by ELISA, and LDH activity was measured using an LDH assay kit.

### Cytotoxicity assay

The cytotoxicity of Cath-HG was determined by the leakage of LDH in platelets. Washed platelets were incubated with Cath-HG (5, 10, and 20 μM) for 5 min at 37 °C and then centrifuged at 12,000× *g* for 5 min at room temperature. Cytotoxicity was determined by measuring the release rate of LDH in the supernatant. 0.1% (*v/v*) Triton X-100 was used to lyse platelets to completely release LDH.

### Platelet aggregation assay

Platelet aggregation was evaluated using PRP. After incubation with Cath-HG (5, 10, and 20 μM) at 37 °C for 5 min, aggregation was initiated by the addition of 10 μM ADP, 10 μg/ml collagen, or 0.6 nM convulxin. Platelet aggregation within 5 min was recorded using an AG400 Platelet Aggregation Analyzer (Techlink, Beijing, China). Platelet-poor plasma and PRP served as the 100% aggregation and 100% aggregation calibration points, respectively.

### Platelet spreading assay

The 14-mm glass coverslips were placed on a 24-well plate and coated overnight with 50 μg/ml collagen or 25 μg/ml fibrinogen, with 1% bovine serum albumin (BSA) serving as a control. The coverslips were then blocked with 1% BSA at room temperature for 1 h and washed with PBS. Washed platelets were incubated with Cath-HG (5, 10, and 20 μM) at 37 °C for 10 min and added to the wells, allowing for 1 h of spreading at 37 °C. After spreading, the coverslips were washed with PBS, and adhered platelets were fixed with paraformaldehyde, permeabilized with 0.1% (*v/v*) Triton X-100, and stained with Alexa Fluor 488-labeled phalloidin. A fluorescence microscope (IX73, Olympus, Tokyo, Japan) was used to obtain images.

### Flow cytometry

To detect whether Cath-HG could bind to platelets, washed platelets were incubated with FITC-labeled Cath-HG (5, 10, and 20 μM) at 37 °C in the dark for 30 min. Samples were then analyzed by flow cytometry (BD Biosciences, CA, USA). To investigate whether Cath-HG acted on α_2_β_1_, washed platelets pretreated with Cath-HG (5, 10, and 20 μM) were incubated with FITC-labeled anti-β_1_ antibody at 37 °C in the dark for 15 min. Samples were then analyzed by flow cytometry (BD Biosciences, CA, USA).

### Western blot

Resting washed platelets or platelets preincubated for 5 min with Cath-HG (5, 10, and 20 μM) were stimulated with collagen for 5 min. The platelets were lysed with radioimmunoprecipitation assay buffer containing protease inhibitor and phosphatase inhibitor cocktails, and the protein concentration was determined with a bicinchoninic acid protein assay kit before SDS-polyacrylamide gel electrophoresis separation and polyvinylidene difluoride (PVDF) membrane transferring. The membranes were incubated with corresponding primary antibodies overnight at 4 °C and appropriate horseradish peroxidase-conjugated secondary antibodies at room temperature for 1 h after being blocked with 5% skimmed milk at room temperature for 1 h. Finally, the results were recorded using a FluorChem R chemiluminescence imaging system (ProteinSimple, CA, USA) and analyzed using ImageJ software.

### Molecular docking

Molecular docking was performed using ZDOCK as previously described [55,56]. In brief, Cath-HG and GPVI (PDB ID: 5OU8) were separately uploaded to ZDOCK 3.0.2 (https://zdock.umassmed.edu/), and receptor–ligand complex models were predicted by rigid docking to investigate the binding site and interaction mode of Cath-HG with GPVI.

### Biotin-based pull-down assay

To identify platelet proteins that bind to Cath-HG, Cath-HG was immobilized on streptavidin resin after labeling with desulfurized biotin according to the manufacturer’s instructions. Subsequently, washed platelet lysate was added to the resin and incubated overnight at 4 °C. On the next day, the resin was washed twice with PBS and resuspended in biotin elution buffer, followed by gentle mixing at 37 °C for 10 min and centrifugation at 500× *g* for 1 min. The resulting solution contains the biotinylated protein and the captured protein, which were analyzed using western blot.

### ELISA-based binding assay

Fifty microliters of Cath-HG (50 to 0.0031 μg/ml) at different concentrations were added to a 96-well plate and incubated at 4 °C overnight. Then, the wells were blocked with 1% BSA at room temperature for 1 h, followed by the addition of 50 μl of washed platelet lysate and incubated at 22 °C for 2 h. After the wells were washed 3 times with PBS with Tween and incubated with anti-GPVI primary antibody at room temperature for 1 h, the wells were then washed again and incubated with corresponding secondary antibodies at room temperature for 1 h. After washing the wells 5 times with PBS with Tween, tetramethylbenzidine substrate was added to visualize horseradish peroxidase enzymatic reaction as the normal procedure and the absorbance at 450 nm was measured with a microplate reader.

### Dot blot

To immobilize Cath-HG, 10 μl of each concentration (0.25, 0.5, 1, and 2 mg/ml) was spotted onto activated PVDF membranes and incubated at 37 °C for 20 min. Following this, the membranes were blocked with 5% BSA and were then incubated with washed platelet lysates at 4 °C overnight. After washing with PBS 3 times, the membranes were incubated with anti-GPVI primary antibody for 6 h at 4 °C, followed by incubation with the corresponding secondary antibody for 1 h and washing TBST. Finally, the results were examined using the FluorChem R chemiluminescence imaging system (ProteinSimple, CA, USA).

### CETSA

Cell lysate CETSA experiment was performed as described by Lang et al [57]. Briefly, platelets were lysed by freezing and thawing in liquid nitrogen for 5 cycles, and the lysate was divided into 2 parts, with one used as the control and the other incubated with Cath-HG (50 μM) at room temperature for 30 min. The lysate was then heated for 3 min at indicated temperatures (37 to 65 °C) and cooled at room temperature. For in-cell CETSA experiment, platelets were treated with Cath-HG (50 μM) for 30 min and then heated for 3 min at indicated temperatures (37 to 65 °C) and cooled at room temperature. The lysate was collected by freezing and thawing in liquid nitrogen for 5 cycles. All lysate supernatants were collected by centrifugation at 12,000× *g* at 4 °C for 10 min, and the contents of GPVI were then analyzed by Western blot.

### FeCl_3_-induced carotid artery thrombosis model

The carotid artery thrombus experiment was carried out as described by Zhang et al [58]. In brief, mice were intravenously injected with 0.9% saline (*w/v*), Cath-HG (5 mg/kg), or heparin (1,000 U/kg) 30 min before surgery. Anesthesia was induced with 2.0% isoflurane before the left carotid artery was exposed through a midline neck incision. A small piece of filter paper (2 mm × 1 mm) saturated with 10% (*w/v*) FeCl_3_ solution was placed on the carotid artery for 5 min to induce thrombus formation. The filter paper was then removed, and the artery was washed with saline. The carotid blood flow and occlusion time were measured to evaluate thrombus formation with the RFLSI Pro+ laser speckle blood flow imaging system (RWD, Shenzhen, China).

### Statistical analysis

All data are presented as mean ± standard deviation (SD), with *n* representing the number of independent experiments. Statistical analysis was made using GraphPad Prism 8 software. One-way analysis of variance (ANOVA) followed by Dunnett's post hoc test was used to compare means. Student *t* test was employed to analyze the statistical significance between 2 groups. *P* < 0.05 was considered to be statistically significant.

## Data Availability

All data needed in the paper are present in the paper and in the Supplementary Materials. Additional data related to this paper may be requested from the authors.
