# Supplementary material for: Cathelicidin-HG Alleviates Sepsis-Induced Platelet Dysfunction by Inhibiting GPVI-Mediated Platelet Activation
Source: Research (Wash D C). 2024 Jun 5;7:0381. doi: 10.34133/research.0381 (PMC11151873; doi:10.34133/research.0381)
Supplement: Supplementary 1 — Figs. S1 to S13 [file research.0381.f1.zip › Supplementary Materials Legends.docx]

Supplementary Materials

**Fig. S1.** Multisequence alignment of cathelicidins from amphibians or frogs.

**Fig. S2.** RP-HPLC and MALDI-TOF-MS spectra of Cath-HG, Cath-HG-1, Cath-HG-2 and Cath-HG-3.

**Fig. S3.** 2D DIPSI spectrum (70 ms mixing time) of Cath-HG (0.8 mM) in 50 mM NaPi buffer (pH 7.0) containing the fingerprint HN-Hα connectivity.

**Fig. S4.** Chemical shift index (CSI) of Cath-HG as determined separately for Hα and Cα resonances in the peptide.

**Fig. S5.** 2D NOESY spectrum (150 ms mixing time) of 0.8 mM Cath-HG in 50 mM NaPi buffer (pH 7.0) and 55 mM SDS-d^25^ recorded at 37°C.

**Fig. S6.** ^13^C-^1^H HSQC spectra of Cath-HG (0.8 mM) displaying the Hα-Cα region (excluding the connectivity of Gly1).

**Fig. S7.** Natural abundance ^15^N-^1^H HSQC spectrum of 0.8 mM Cath-HG in 50 mM NaPi buffer (pH 7.0) with no additives.

**Fig. S8.** The concentrations of pro-inflammatory cytokines in serum measured by ELISA.

**Fig. S9.** Effect of Cath-HG on neutrophil activation in septic mice.

**Fig. S10.** Cathelicidins from other amphibians have no inhibitory effect on collagen-induced platelet aggregation.

**Fig. S11.** Effect of Cath-HG on GPVI downstream signaling pathways.

**Fig. S12.** Representative images of histopathological analysis of lung, liver, and kidney stained with H&E at 24 h after Cath-HG (5 mg/kg) was injected intravenously into mice 2 h after CLP.

**Fig. S13.** Toxicity of Cath-HG *in vivo*.
