# Supplementary figures and images for: Cathelicidin-HG Alleviates Sepsis-Induced Platelet Dysfunction by Inhibiting GPVI-Mediated Platelet Activation
Source: Research (Wash D C). 2024 Jun 5;7:0381. doi: 10.34133/research.0381 (PMC11151873; doi:10.34133/research.0381)

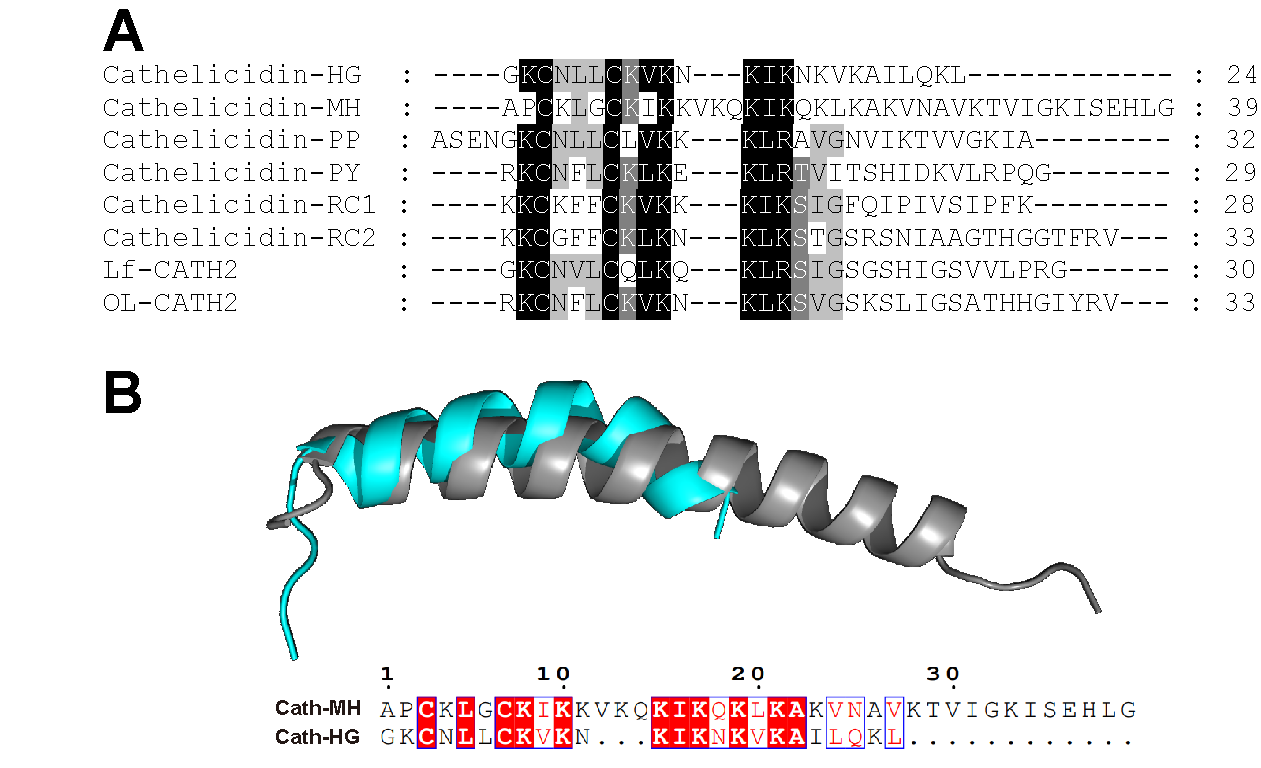

Supplement: Supplementary 1 — Figs. S1 to S13 [file research.0381.f1.zip › FigS1.tif]

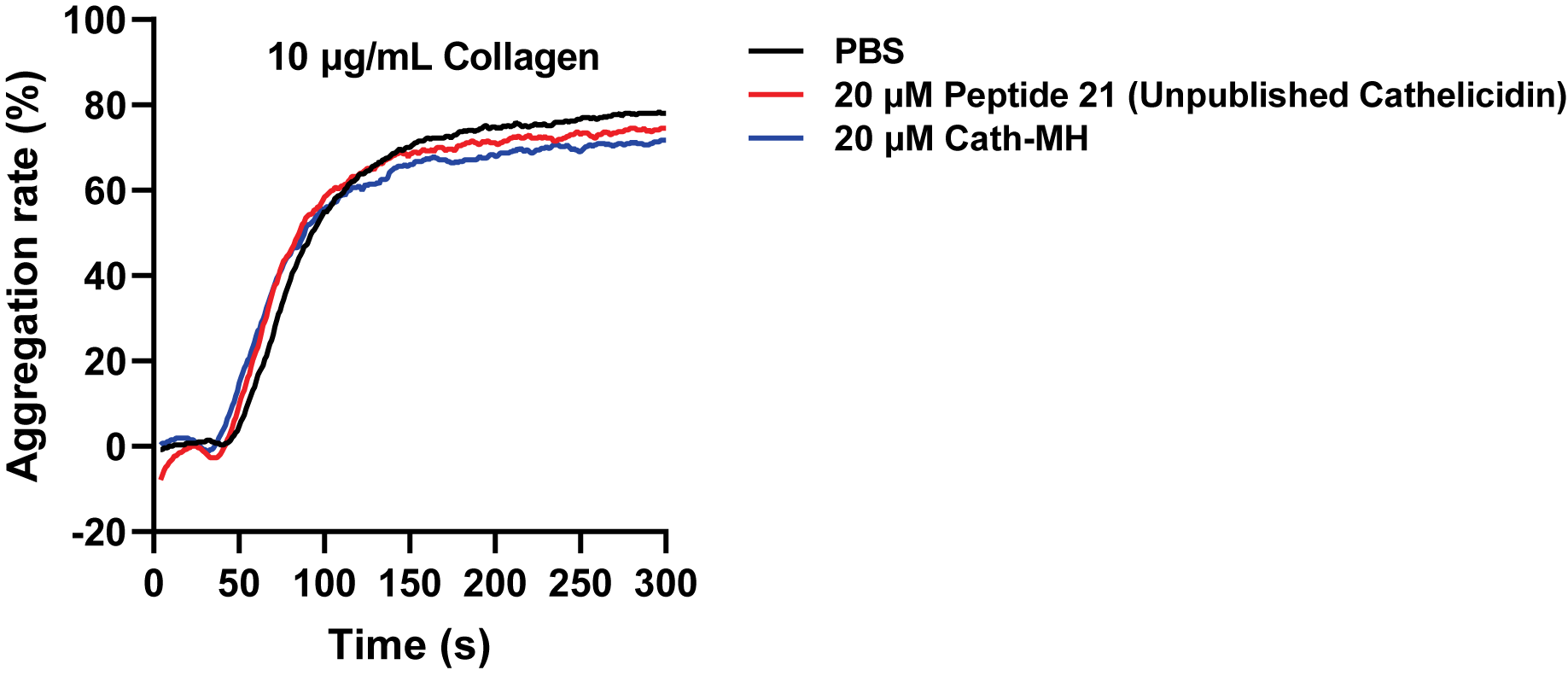

Supplement: Supplementary 1 — Figs. S1 to S13 [file research.0381.f1.zip › FigS10.tif]

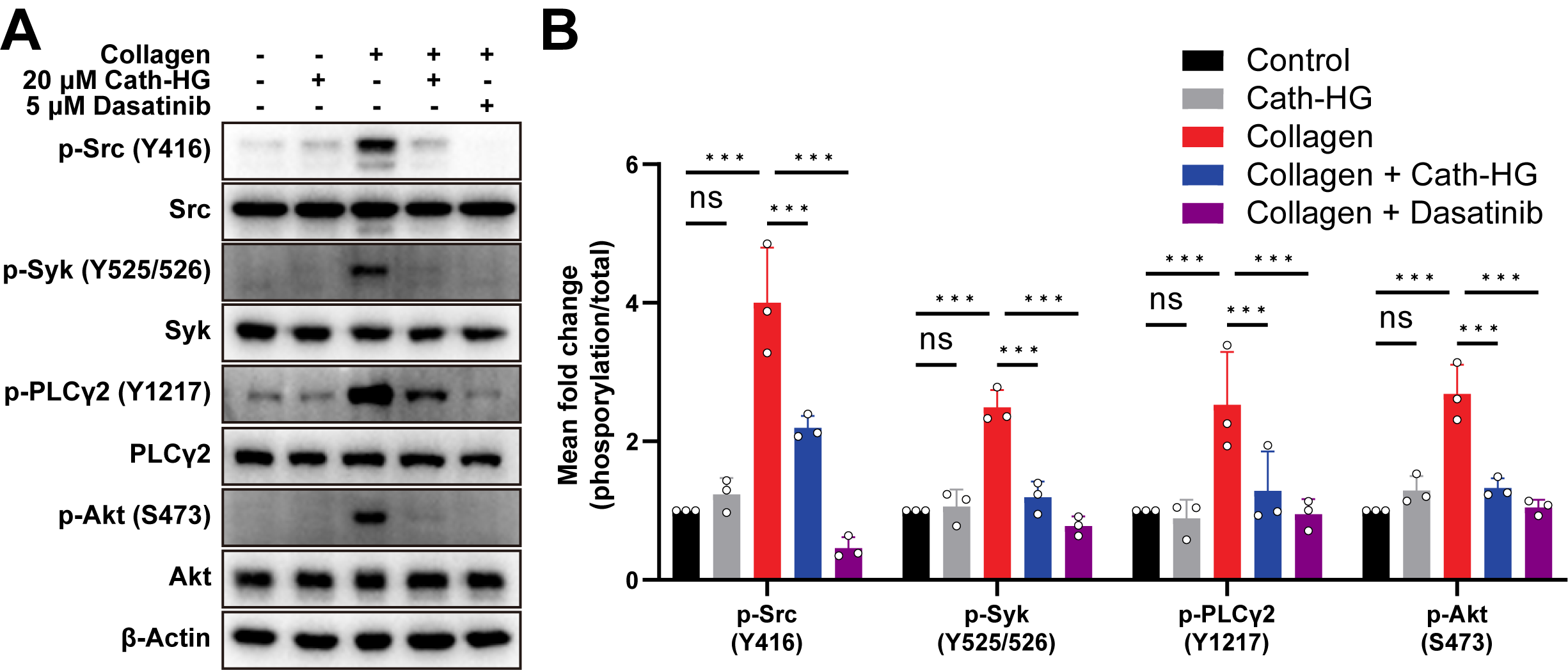

Supplement: Supplementary 1 — Figs. S1 to S13 [file research.0381.f1.zip › FigS11.tif]

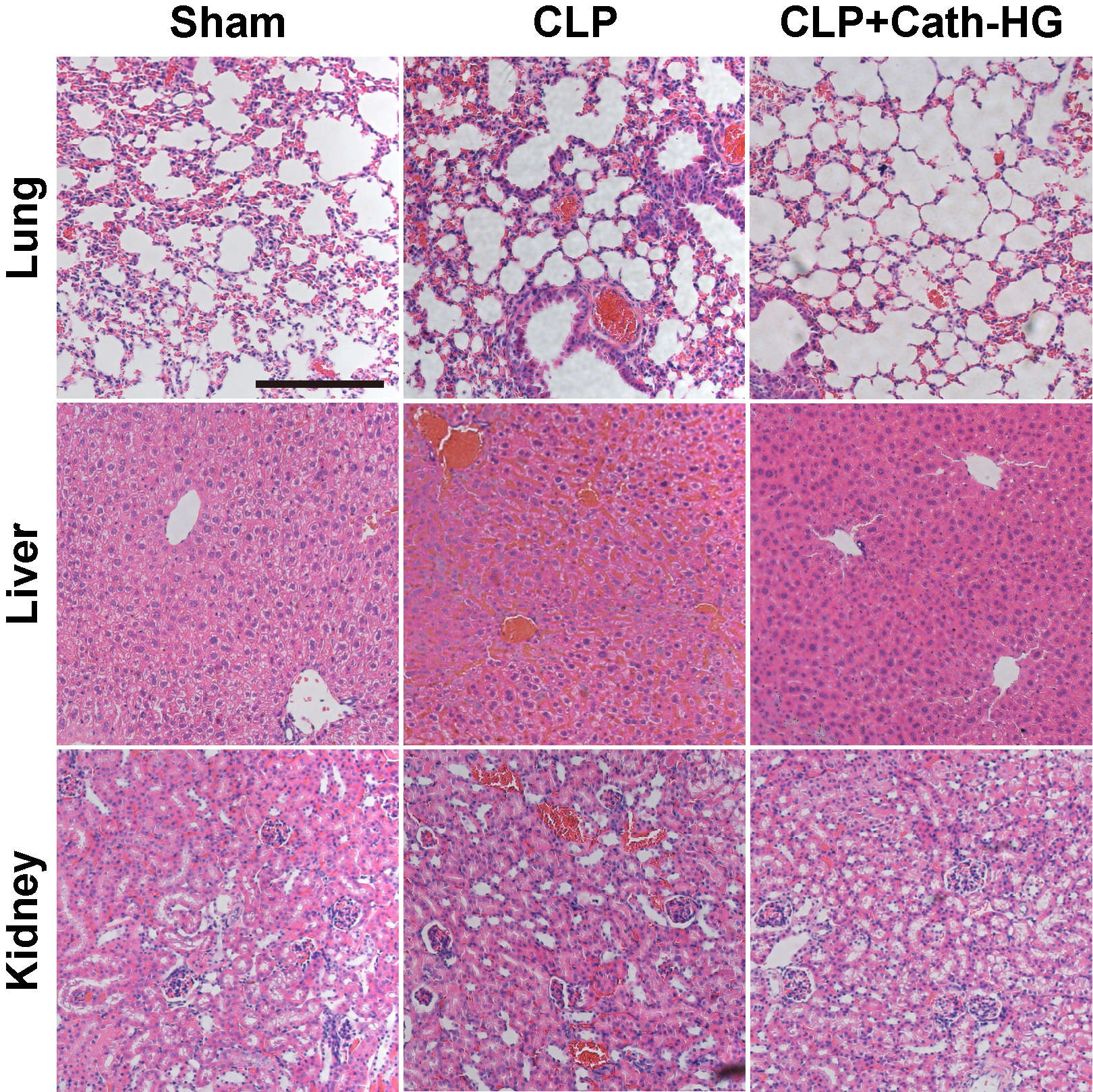

Supplement: Supplementary 1 — Figs. S1 to S13 [file research.0381.f1.zip › FigS12.tif]

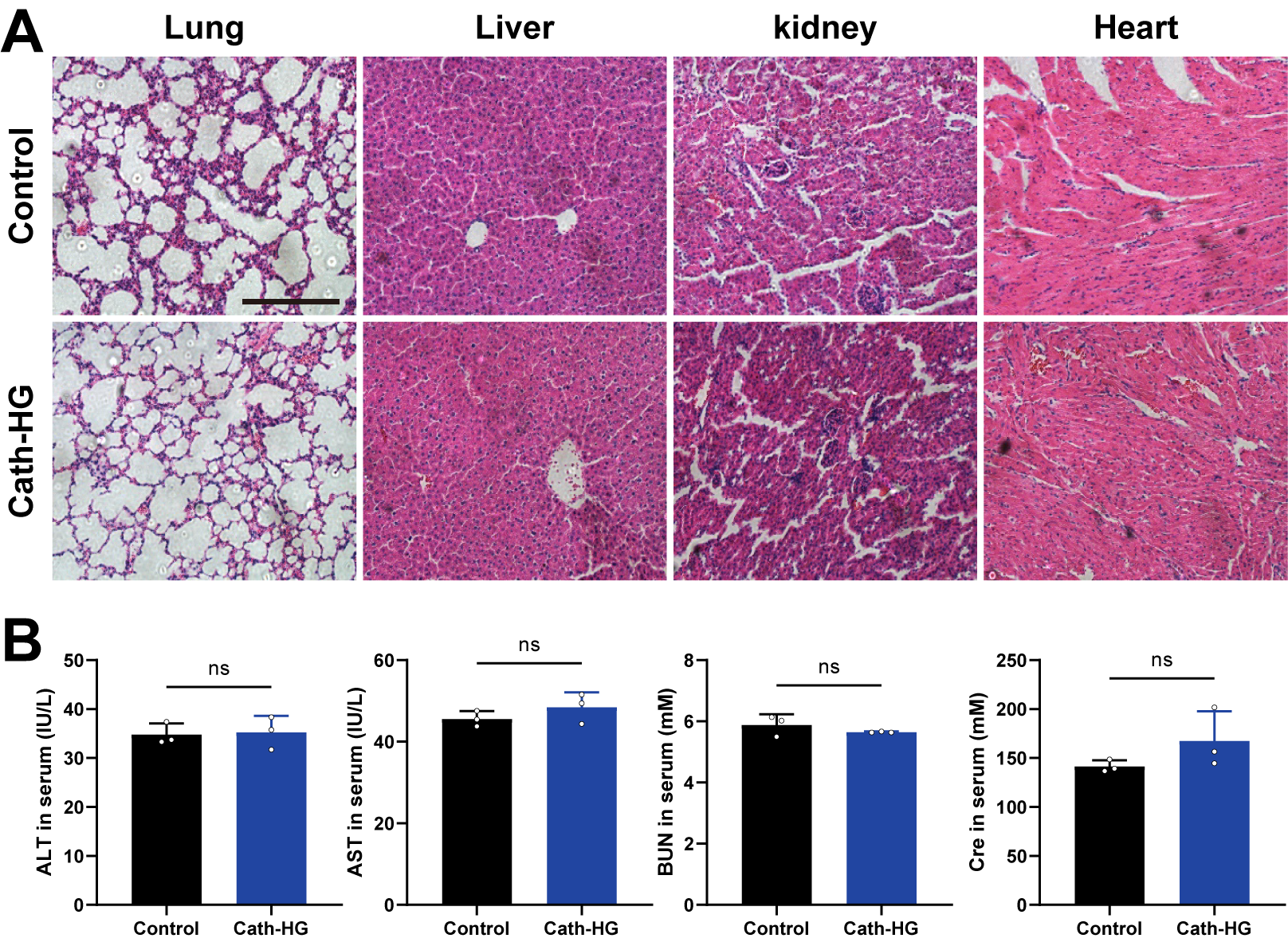

Supplement: Supplementary 1 — Figs. S1 to S13 [file research.0381.f1.zip › FigS13.tif]

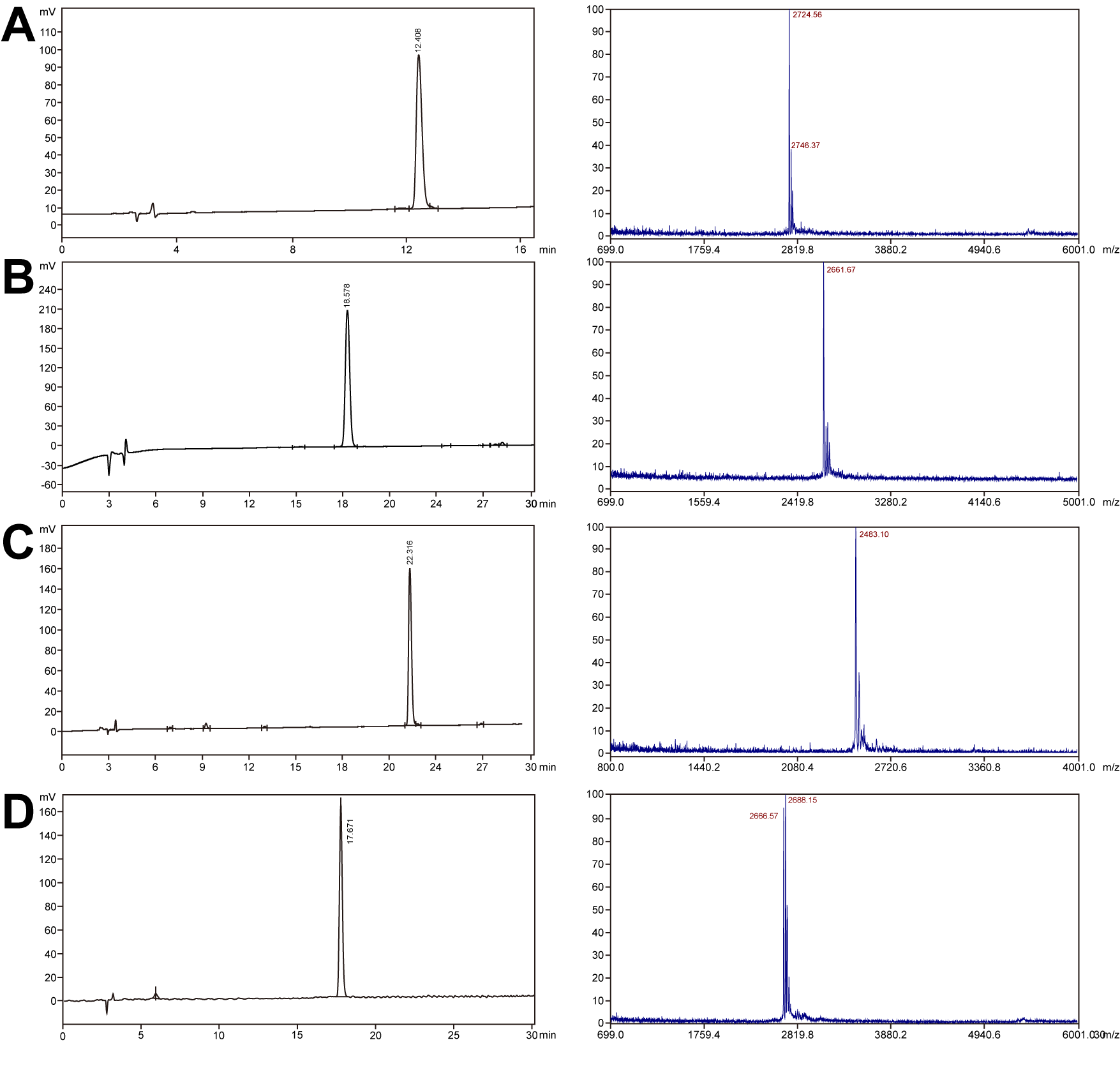

Supplement: Supplementary 1 — Figs. S1 to S13 [file research.0381.f1.zip › FigS2.tif]

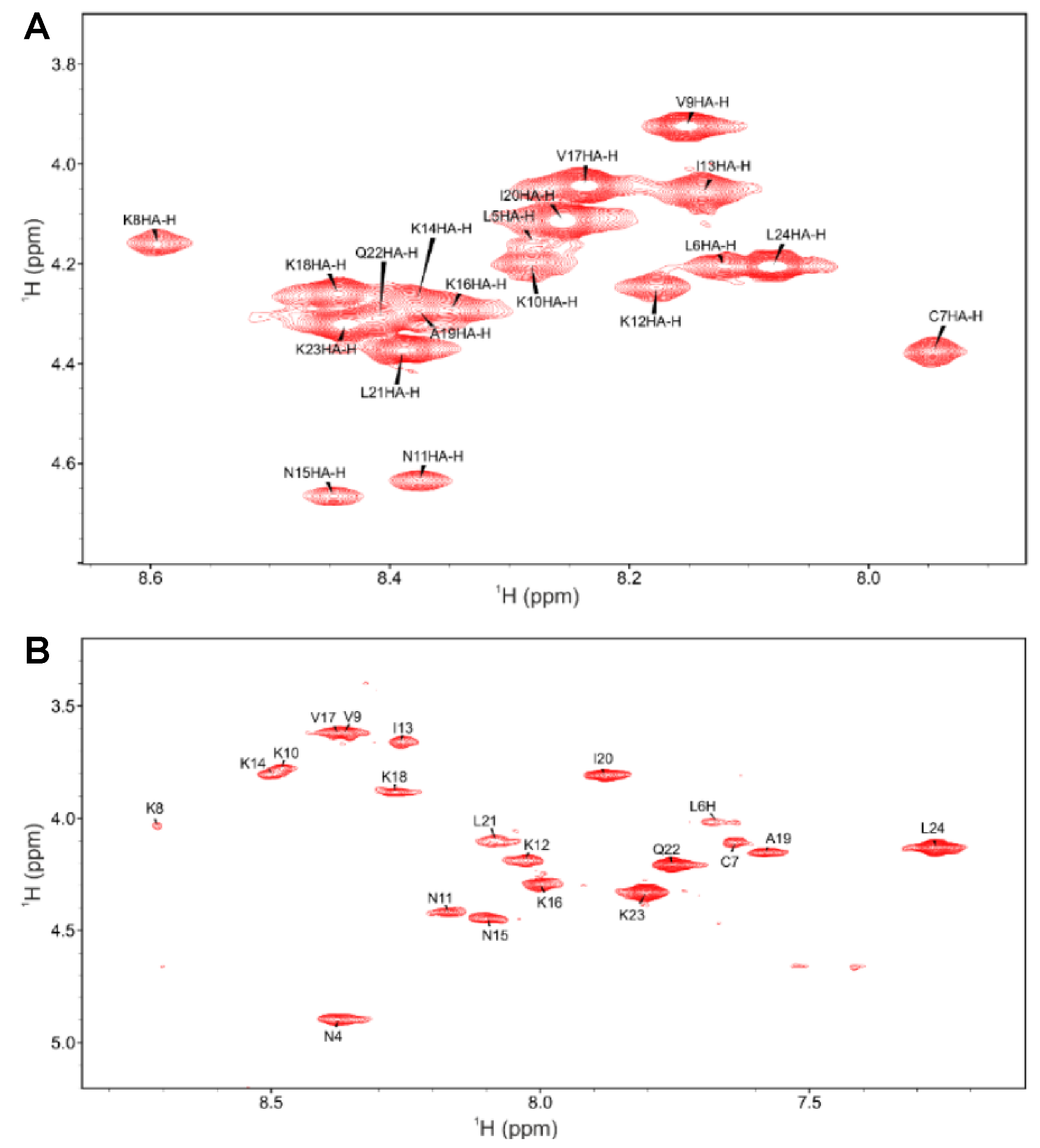

Supplement: Supplementary 1 — Figs. S1 to S13 [file research.0381.f1.zip › FigS3.tif]

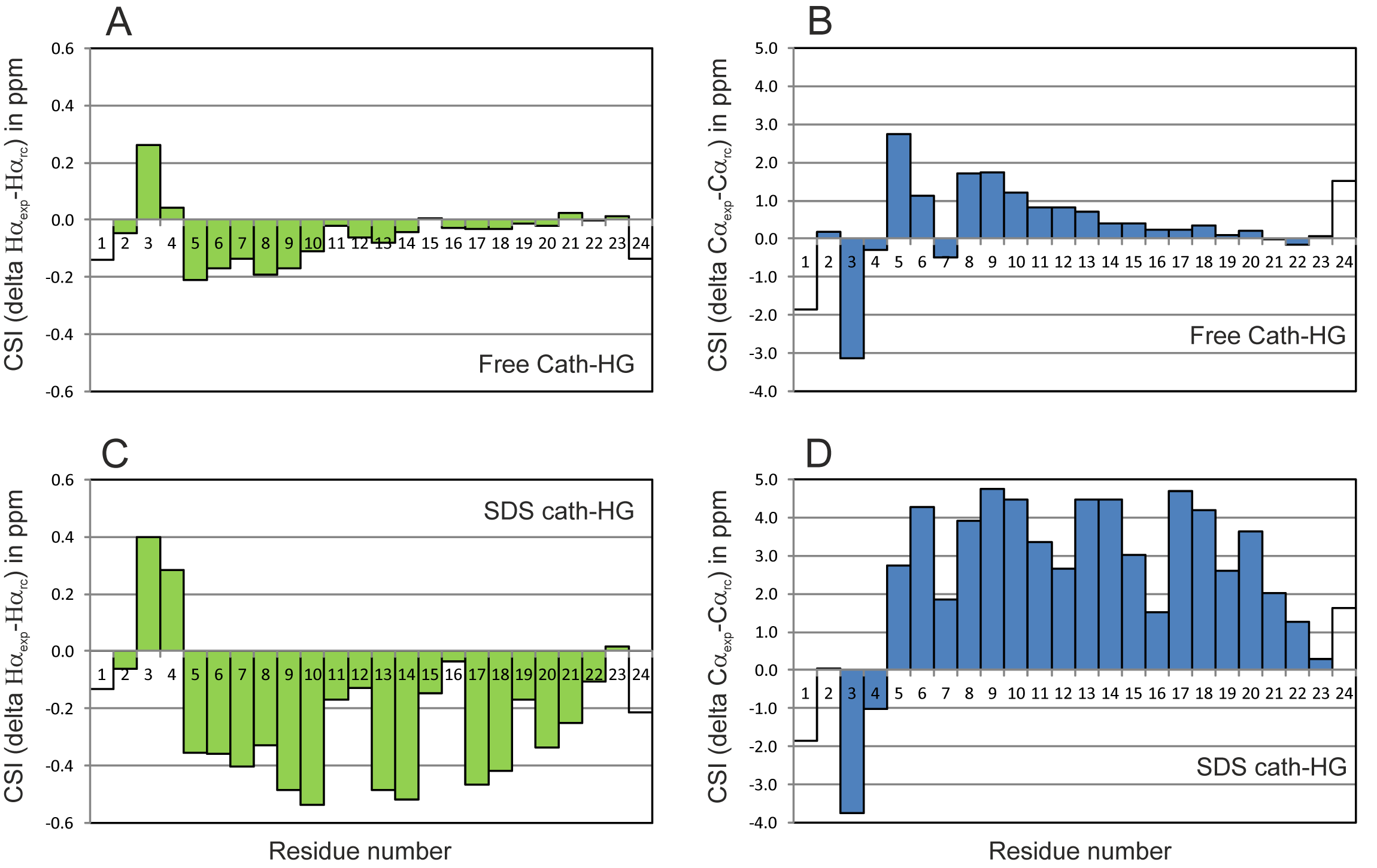

Supplement: Supplementary 1 — Figs. S1 to S13 [file research.0381.f1.zip › FigS4.tif]

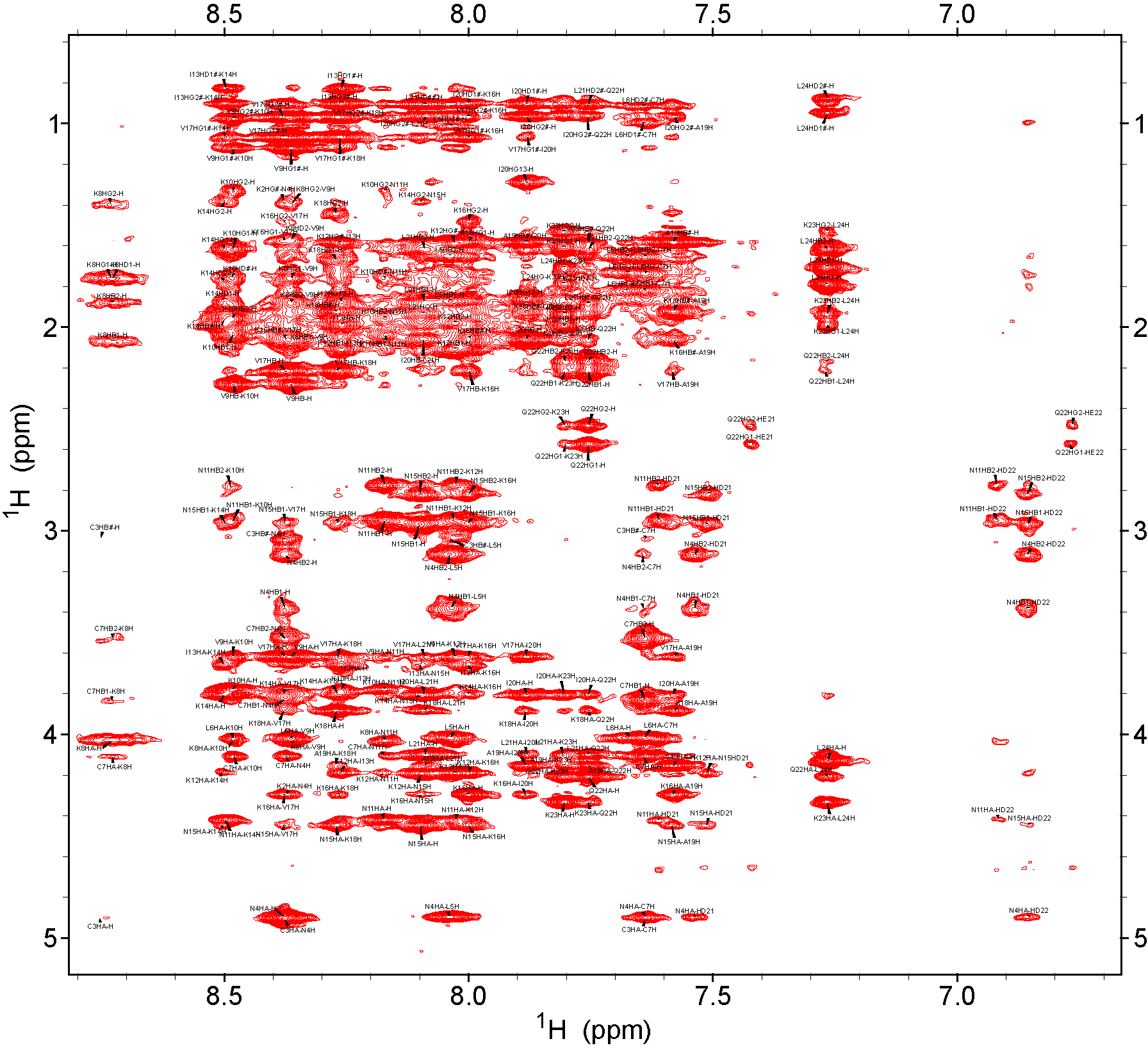

Supplement: Supplementary 1 — Figs. S1 to S13 [file research.0381.f1.zip › FigS5.tif]

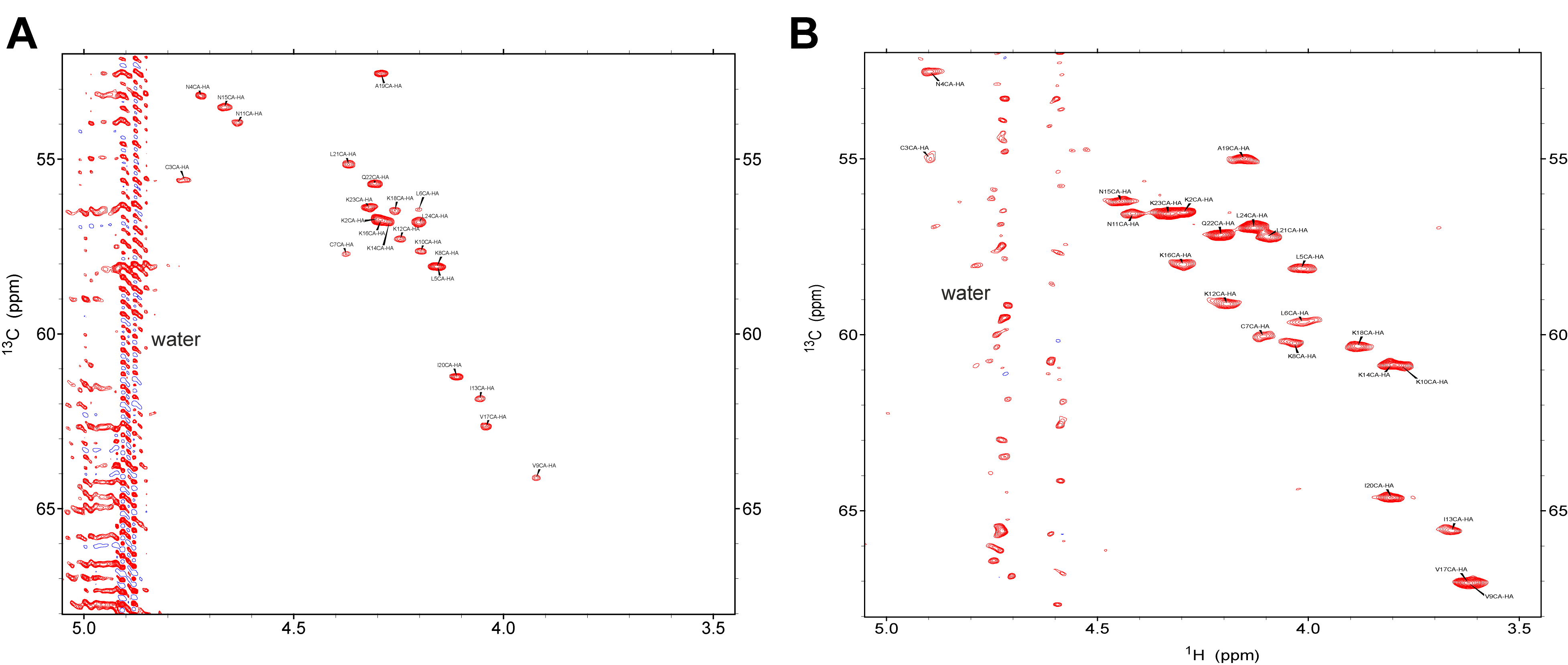

Supplement: Supplementary 1 — Figs. S1 to S13 [file research.0381.f1.zip › FigS6.tif]

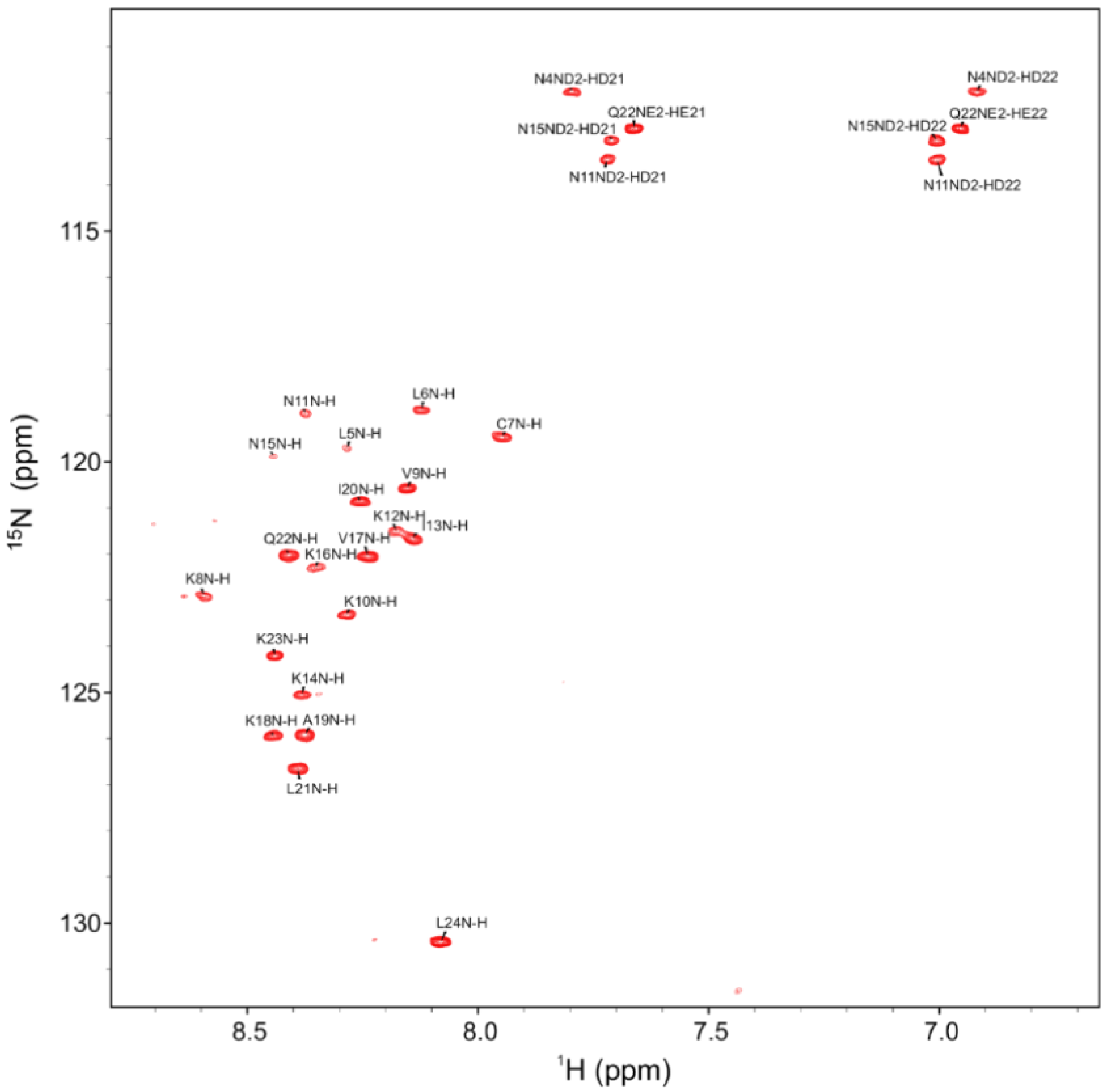

Supplement: Supplementary 1 — Figs. S1 to S13 [file research.0381.f1.zip › FigS7.tif]

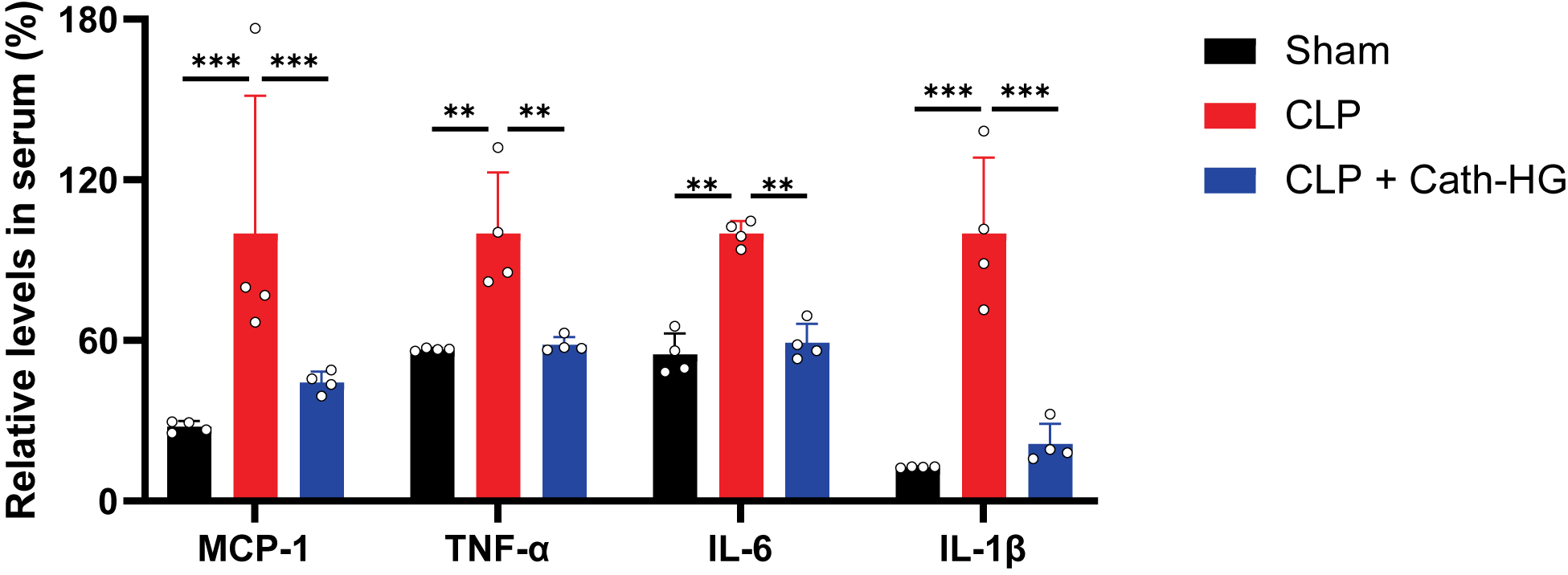

Supplement: Supplementary 1 — Figs. S1 to S13 [file research.0381.f1.zip › FigS8.tif]

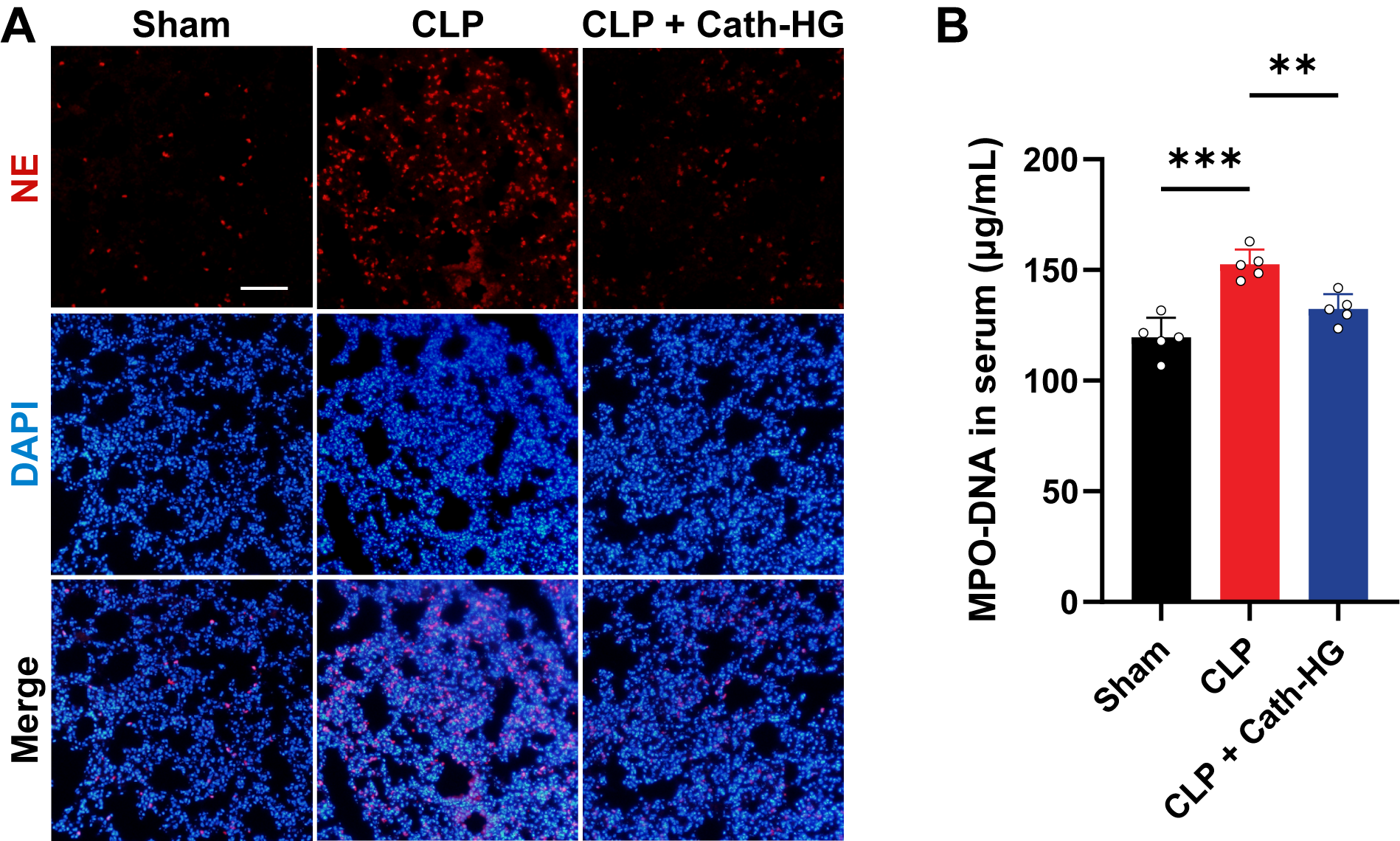

Supplement: Supplementary 1 — Figs. S1 to S13 [file research.0381.f1.zip › FigS9.tif]
